# PPDRTL: A novel framework for predicting pollutant deposition on roadside tree leaves using linear regression

**DOI:** 10.1371/journal.pone.0326588

**Published:** 2026-09-08

**Authors:** Neelam Yadav, Sunil K. Singh, Dinesh Sharma, Sudhakar Kumar, Varsha Arya, Wadee Alhalabi, Hind Alsharif, Brij Bhooshan Gupta, Kwok Tai Chui

**Affiliations:** 1 Computer Science and Engineering, Chandigarh College of Engineering and Technology, Chandigarh, India; 2 Electronics and Communication Engineering, Chandigarh College of Engineering and Technology, Chandigarh, India; 3 Electronic Engineering and Computer Science, Hong Kong Metropolitan University, Hong Kong, China; 4 Interdisciplinary Research, University of Petroleum and Energy Studies (UPES), Dehradun, India; 5 Department of Computer Science, Immersive Virtual Reality Research Group, King Abdulaziz University, Jeddah, Saudi Arabia; 6 Computer Science and Artificial Intelligence department, College of Computing, Umm Al-Qura University, Makkah, Saudi Arabia; 7 Department of Computer Science and Information Engineering, Asia University, Taichung, Taiwan; 8 Department of Medical Research, China Medical University Hospital, China Medical University, Taichung, Taiwan; 9 Symbiosis Centre for Information Technology (SCIT), Symbiosis International University, Pune, India; 10 School of Cybersecurity, Korea University, Seoul, South Korea; Southeast University, CHINA

## Abstract

Air quality plays a critical role in human well-being, as air pollution significantly contributes to respiratory diseases such as pneumonia, cysts, and asthma. Predicting pollution levels can enable targeted interventions to mitigate associated chronic health risks. According to the World Health Organization, outdoor air pollution is responsible for approximately 4.2 million premature deaths worldwide. This paper proposes a PPDRTL framework to predict roadside air quality based on pollutant deposition on tree leaves. High-resolution images of roadside leaves are captured and analyzed to estimate pollution levels at the street scale. Image features, including contrast, entropy, and standard deviation, are extracted under varying traffic conditions, namely high, medium, and low traffic densities. Segmentation techniques such as PSO, DPSO, and FODPSO are employed to enhance pollutant feature extraction from leaf surfaces. The PPDRTL framework utilizes a Linear Regression model to predict air quality index (AQI) values from leaf image features, while ground truth data are obtained from the Haryana State Pollution Control Board (HSPCB) for validation. For the 10-day pilot dataset, FODPSO-based PPDRTL achieves *R*^2^ up to 0.894, with RMSE in the range of $8.76-12.34\;\mu g/m^{3}$ across high-, medium-, and low-traffic sites. Furthermore, the framework achieves prediction accuracies of 88.91% for high-traffic areas, 90.70% for medium-traffic areas, and 91.42% for low-traffic areas, demonstrating its effectiveness as a robust approach for fine-grained air quality prediction and environmental monitoring.

## 1. Introduction

Rapid socio-economic development, coupled with accelerating urbanization and industrialization, has led to a significant deterioration in ambient air quality worldwide. The growth in population has resulted in increased automobile usage, contributing to higher vehicular emissions that adversely affect roadside vegetation and urban air environments [1].

Globally, air pollution has emerged as a major environmental and public health concern, with nearly 90% of the population residing in areas where pollution levels exceed the limits prescribed by the World Health Organization (WHO) [2]. Key contributors include anthropogenic activities such as industrial production, vehicular emissions, and coal combustion, which release particulate matter and harmful gaseous pollutants into the atmosphere [3]. Consequently, poor air quality has become one of the leading risk factors for mortality worldwide [4], significantly impacting overall environmental and human health [5].

Plants serve as effective bio-indicators for predicting and measuring air pollution levels. By monitoring plant health and leaf conditions, researchers can infer the presence and severity of pollutants because specific plant species are sensitive to particular contaminants. In India, pollutant concentrations are measured through 703 monitoring stations, and the Air Pollution Tolerance Index (APTI) is used to evaluate plant responses based on four biochemical leaf characteristics [6]. Research indicates that chlorophyll degradation, which results in leaf yellowing, reduces photosynthetic capability in plants exposed to pollution [7]. Several studies have examined dust deposition on roadside plants to assess changes in epidermal characteristics, pigment concentration, and carbon and nitrogen content [8,9]. Techniques such as spectrophotometry and liquid chromatography help quantify plant pigment alterations [10]. Additionally, dust pollution induces physiological changes, including stretched epidermal cells, malformed stomata, and plasmolysis of guard cells, and it reduces plant conductance indices (PCI) [11]. Leaf macro-morphological traits (LMTs) of urban trees also provide insight into particulate matter (PM) retention [12,13]. Strong correlations have been reported between leaf chlorophyll content and spectral properties, supporting the use of spectroscopy for early detection of plant stress [14]. Studies using APTI have further evaluated seasonal variation in the sensitivity of species such as *Polyalthia longifolia*, *Swietenia mahagoni*, and *Artocarpus heterophyllus* [15].

Although India’s 703 air quality monitoring stations provide valuable information, they assess pollution only at specific locations and do not capture street-level variations that are essential for understanding commuter exposure. Rising population density and increased vehicular usage highlight the need for continuous pollution monitoring across high-, medium-, and low-traffic areas. Existing systems also lack real-time prediction of pollutant components, which limits proactive air-quality management and increases the risk of chronic diseases for daily travelers.

Despite extensive work on plant-based bioindicators, several critical research gaps persist. First, existing monitoring infrastructure does not provide fine-grained, street-level pollution data. Second, prior studies primarily rely on biochemical indices such as APTI, pigment content, and chlorophyll loss, rather than automated and scalable image-based prediction systems. Third, comparative analyses across traffic density categories remain limited, even though traffic volume strongly influences pollutant deposition patterns. Fourth, optimized image-segmentation techniques, including PSO, DPSO, and FODPSO, have not been systematically integrated with machine learning for pollution prediction from leaf-surface features. Finally, very few studies offer statistically validated and continuous prediction frameworks capable of estimating fine particulate matter using high-resolution leaf images.

Motivated by these gaps, this work proposes the Predicting Pollutant Deposition on Roadside Tree Leaves (PPDRTL) framework, which estimates roadside PM_2.5_ concentrations by linking image-derived texture features from segmented leaf regions to regulatory PM_2.5_ measurements. High-resolution RGB images of roadside tree leaves are preprocessed using wavelet-based denoising, segmented with PSO/DPSO/FODPSO to isolate pollutant-affected regions, and converted into digital-number (DN) features such as contrast, entropy, and standard deviation. These features are then mapped to official NAQI/HSPCB PM_2.5_ values using linear regression, thereby providing a practical, image-based extension of existing station data to nearby street segments.

The specific objectives of this study are:

- To develop the PPDRTL framework for predicting PM_2.5_ from roadside tree leaf images.
- To quantify relationships between DN-based texture features (contrast, entropy, standard deviation) and pollutant deposition on leaves.
- To evaluate the effectiveness of PSO-, DPSO-, and FODPSO-based segmentation within PPDRTL by analyzing their correlations with AQI values from HSPCB using linear regression.

## 2. Literature review

Early studies introduced the Air Pollution Tolerance Index (APTI) to evaluate plant tolerance based on physiological parameters [16]. Subsequent work showed that air pollutants, particularly dust deposition, alter leaf characteristics such as pigment content, chlorophyll concentration, and reflectance properties [17,18]. Several ecological studies have assessed plant sensitivity to pollution by analyzing biochemical, physiological, and morphological traits across multiple species and environments [19,20]. These studies establish that roadside vegetation responds measurably to pollution, but they often depend on destructive sampling and laboratory instruments, which limits their suitability for dense, street-level monitoring.

In parallel, machine learning techniques such as gradient boosting, random forests, linear regression, and artificial neural networks have been applied to predict environmental variables, including *PM*_2.5_ concentrations and weather conditions [21,22]. Hyperspectral and imaging-based studies demonstrate strong correlations between leaf reflectance, spectral indices, dust accumulation, and associated biochemical stress, showing that leaf optical and textural properties can encode information about deposited particulates [23–26]. Additional approaches employ RGB imaging, scanning electron microscopy (SEM), and hyperspectral sensing to analyze pollution-induced changes in vegetation and explore image-based particulate characterization [27]. Together, these works indicate that supervised models can learn quantitative mappings from spectral or image features to environmental variables.

As summarized in Table 1, most existing approaches, although powerful, rely on specialized instruments or laboratory-based analysis and are primarily designed for leaf-level dust quantification rather than operational, street-level air-quality prediction. They rarely provide a unified framework that directly links easily acquired RGB images of roadside vegetation to regulatory PM_2.5_ measurements.

**Table 1:** Comparison of image/spectroscopy-based approaches for pollution assessment vs. the proposed PPDRTL framework.

Criteria	Zhu et al. (2020) [23]	Zhu et al. (2021) [25]	Rai and Panda (2014) [26]	Zhu et al. (2019) [24]	Tiwari et al. (2024) [27]	PPDRTL (This Work)
**Sensing modality**	Leaf hyperspectral reflectance (350–2500 nm) under simulated dust loads	Leaf hyperspectral reflectance plus functional traits	Macroscopic leaf imaging plus biochemical assays	Leaf hyperspectral imaging under pollution gradients	Multiple modalities: RGB, SEM, hyperspectral imaging	High-resolution RGB leaf images with wavelet preprocessing (DWT + SWT) and metaheuristic segmentation
**Targets**	Leaf dust retention and spectral changes	Dust deposition and chlorophyll variation	Dust deposition and biochemical plant responses	Dust deposition prediction using spectral responses	Image-based particulate matter characterization	Roadside PM_2.5_ prediction using DN-based leaf features
**Spatial scale**	Leaf-level controlled experiment	Leaf-level across urban environments	Roadside leaf-level monitoring	Leaf-level pollution gradient studies	Microscopic to urban scale depending on study	Street-level monitoring across roadside sites in Gurugram
**Tree species / vegetation**	*Euonymus japonicus*	Urban plant species used as environmental indicators	Species such as *Polyalthia longifolia*, *Azadirachta indica*	Urban ornamental species	Various plant species summarized	*Alstonia scholaris*, *Millettia pinnata*, *Alstonia macrophylla*
**Method**	Spectral feature analysis with regression modelling	Hyperspectral indices combined with functional trait analysis	Gravimetric dust measurement and Air Pollution Tolerance Index (APTI)	Spectral-based dust deposition prediction models	Review of thresholding, ML and object detection for PM image analysis	Wavelet preprocessing + PSO/DPSO/FODPSO segmentation + DN feature regression
**Reported metrics**	Correlation between dust load and spectral reflectance (*R*^2^)	Best model $R^{2}\approx 0.787$	APTI scores and biochemical correlations	Spectral–dust correlation models	Survey of accuracies reported in PM imaging studies	*R*^2^ up to 0.894 with RMSE ≈ 8.76–12.34 $\mu g/m^{3}$
**Key limitations**	Simulated laboratory dust conditions	Requires spectrometer and leaf trait measurements	Destructive biochemical sampling	Focus on dust deposition rather than ambient PM_2.5_	Narrative review without unified prediction framework	Pilot-scale dataset; meteorological variables not integrated
**Novelty / Key Contribution**	Spectral analysis of dust deposition	Hyperspectral trait modeling of dust load	Biochemical tolerance assessment of plants	Spectral dust deposition prediction model	Survey of image-based PM analysis methods	**Traffic-category PM**_**2.5**_ **prediction using RGB leaf images with wavelet preprocessing and metaheuristic segmentation (PSO/DPSO/FODPSO)**

Building on these insights, the primary objective of this study is to estimate PM_2.5_ concentrations by integrating wavelet-based image preprocessing, metaheuristic image segmentation using PSO, DPSO, and FODPSO, digital number (DN)-based feature extraction in the RGB space, and linear regression modeling. Wavelet-based preprocessing is employed because discrete and stationary wavelet transforms effectively denoise images while preserving multiscale texture information that is sensitive to dust deposition and leaf spectral variations [23–25]. Metaheuristic segmentation techniques are preferred over conventional global thresholding because they can optimize Otsu-like criteria in complex and noisy histograms and have been successfully applied in particulate imaging tasks, despite higher computational cost and sensitivity to parameter settings [27]. DN-based RGB texture features such as contrast, entropy, and standard deviation are then extracted as compact descriptors of leaf surface alterations associated with particulate accumulation, consistent with prior work relating image-based and spectral indices to pollution-induced changes in vegetation [26]. Linear regression is used as an interpretable baseline model to map these features to PM_2.5_ concentrations, aligning with earlier environmental modeling studies that employed regression and related machine-learning methods for *PM*_2.5_ and weather prediction [21,22]. Jain et al. 2025 [28] proposed the model for potato blight detection using fuzzy image enhancement. Wang et al. 2020 [29] proposed image

On this basis, the proposed PPDRTL framework utilizes street-level RGB imaging combined with DWT/SWT-based preprocessing and PSO/DPSO/FODPSO segmentation to isolate pollutant-affected regions on leaf surfaces. The extracted DN-based texture and intensity features are correlated with official NAQI and HSPCB PM_2.5_ measurements under varying traffic conditions, yielding a scalable, image-driven methodology for roadside pollution estimation using widely available camera sensors. In contrast to deep learning segmentation approaches such as U-Net and its variants, which require large annotated datasets and substantial training effort [30,31], the PPDRTL segmentation pipeline is training-free and builds on metaheuristic thresholding methods reported in prior PM imaging studies [27], making it more suitable for rapid deployment in data-scarce roadside environments.

## 3. Methodology

**Fig 1:**
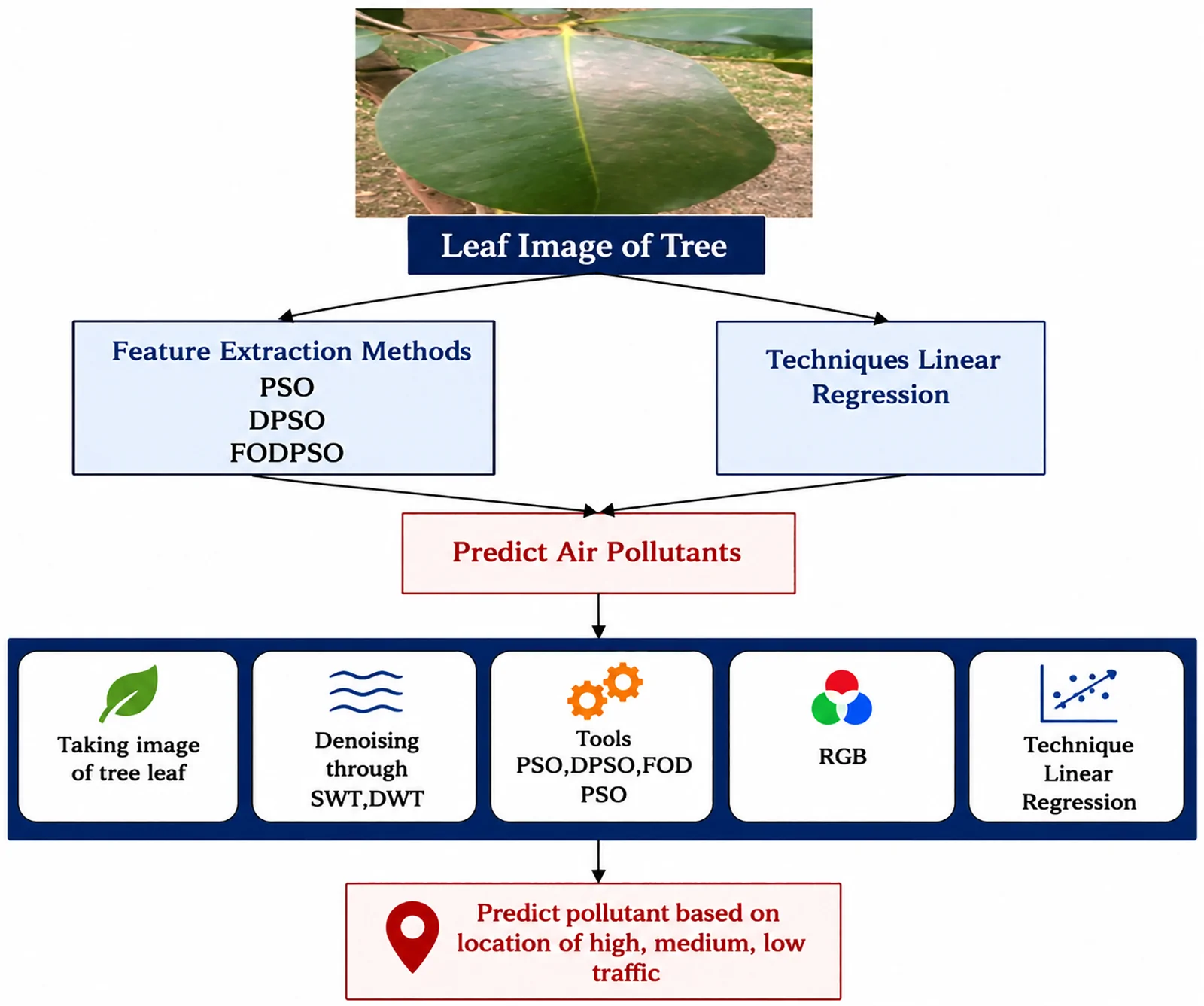
Proposed PPDRTL framework using a linear regression algorithm for PM_2.5_ prediction.

This section presents the PPDRTL framework developed for predicting air pollutants from roadside tree leaves. The overall goal is to estimate PM_2.5_ concentrations by combining wavelet-based image preprocessing, metaheuristic image segmentation (PSO, DPSO, FODPSO), digital number (DN) feature extraction in the RGB space, and linear regression. The overview of the proposed PPDRTL framework is shown in Fig 1. Leaf images are first preprocessed using discrete wavelet transform (DWT) and stationary wavelet transform (SWT); PSO, DPSO, and FODPSO are then used to segment pollutant-affected regions, from which contrast, entropy, and standard deviation are extracted and supplied to a linear regression model together with reference air quality measurements.

### 3.1 Image acquisition protocol

To ensure consistency and comparability of digital number (DN) values across all leaf images, a standardized image acquisition protocol was followed during data collection.

All images were captured using a fixed camera setup with controlled parameters. The camera was operated in manual mode with constant ISO (ISO 100), fixed aperture (f/8), and fixed shutter speed to maintain uniform exposure conditions across different traffic locations. Automatic exposure and white balance adjustments were disabled to avoid variability in pixel intensity values.

Leaf images were captured at a consistent distance of approximately 30–40 cm from the camera sensor, with the camera oriented perpendicular to the leaf surface to minimize geometric distortions. All images were acquired under stable daylight conditions, avoiding harsh shadows and direct sunlight to ensure uniform illumination.

To maintain radiometric consistency, a neutral reference surface (gray card) was included periodically during image acquisition to account for illumination variations. This enabled normalization of DN values across different sessions.

The images were stored in high-resolution RGB format without compression to preserve pixel-level information. These controlled acquisition settings ensure that variations in extracted DN features are primarily due to pollutant deposition rather than imaging inconsistencies.

### 3.2 Wavelet-based preprocessing and DN feature extraction

High-resolution RGB images of roadside tree leaves are first denoised and enhanced using DWT and SWT to remove unwanted noise while preserving edges and texture details [32]. Dilations and shifts of a single mother wavelet Ψ(x) are used to construct the wavelet family:


$$\begin{array}{c} \Psi_{c,d}(x)=\frac{1}{\sqrt{c}}\;\Psi \left(\frac{x-d}{c}\right), \end{array}$$ (1)

where *c* is the scaling factor and *d* is the shifting variable. The combined preprocessing and feature extraction workflow is summarized in Algorithm 1, where Wiener filtering, single-level DWT, and SWT are applied to obtain an enhanced preprocessed image *I*_pre_, and DN-based features are then computed from the segmented leaf region.

In this study, the Daubechies wavelet of order 4 (db4) was selected as the mother wavelet due to its suitability for capturing localized variations and texture details in leaf images. A single-level decomposition was employed for both DWT and SWT to balance noise reduction and computational efficiency. This choice was found sufficient to capture dominant texture variations without introducing redundancy from higher-level decompositions.

During preprocessing, thresholding is implicitly controlled through wavelet coefficient reconstruction rather than explicit hard or soft thresholding, ensuring preservation of important structural details. The combination of Wiener filtering, DWT, and SWT provides effective denoising while maintaining edge and texture information critical for segmentation.

To evaluate the impact of wavelet-based preprocessing, an ablation analysis was conducted by comparing model performance with and without preprocessing (see Table 6). The results showed that the inclusion of DWT and SWT led to improved feature quality, resulting in lower RMSE and higher *R*^2^ values in the regression stage, confirming the effectiveness of the proposed preprocessing pipeline.

**Algorithm 1. Preprocessing and DN feature extraction from segmented leaf region**

**Require:** RGB leaf image *I*_rgb_, segmentation mask *S*, mother wavelet ψ (db4), decomposition level *L* = 1

**Ensure:** Feature vector *F* = [Contrast, Entropy, Std, *R*, *G*, *B*]

1: Convert *I*_rgb_ to grayscale to obtain *I*

2: Apply Wiener filter to *I* to obtain $I_{f}$

3: Perform single-level DWT on $I_{f}$ using ψ and level *L* to obtain (*cA*, *cH*, *cV*, *cD*)

4: Reconstruct denoised image *I*_dwt_ from (*cA*, *cH*, *cV*, *cD*) using inverse DWT

5: Perform single-level SWT on *I*_dwt_ using ψ and level *L* to obtain (*sA*, *sH*, *sV*, *sD*)

6: Reconstruct enhanced image *I*_pre_ from (*sA*, *sH*, *sV*, *sD*) using inverse SWT

7: Extract leaf pixels: *P* = *I*_pre_(*S* = 1)

8: Compute gray-level co-occurrence matrix (GLCM) from *P*

9: Compute **Contrast** from the GLCM

10: Compute **Entropy** of *P*

11: Compute **Std** (standard deviation) of *P*

12: Compute mean RGB intensities *R*, *G*, *B* over pixels of *I*_rgb_ inside mask *S*

13: **return** *F* = [Contrast, Entropy, Std, *R*, *G*, *B*]

### 3.3 Segmentation using PSO, DPSO, and FODPSO

To isolate pollutant deposits on leaf surfaces, three metaheuristic segmentation methods are employed: Particle Swarm Optimization (PSO), Differential/Darwinian PSO (DPSO), and Fractional Order DPSO (FODPSO). These algorithms are applied in a unified way across all traffic categories; only the input leaf images differ (high-, medium-, or low-traffic sites). All three methods share the same Otsu-based fitness function but differ in how particle positions are updated.

#### 3.3.1 Particle Swarm Optimization (PSO).

Particle Swarm Optimization (PSO), proposed by Kennedy and Eberhart [33], is a versatile population-based search strategy that solves a range of optimization problems. A swarm of particles is randomly scattered in the search space, and each particle updates its position and velocity based on its own best experience and that of the swarm. For a particle with position *Y*(*x*) and velocity *Z*(*x*) at iteration *x*, the updates are:


$$\begin{array}{c} Y(x+1)=Y(x)+Z(x+1), \end{array}$$ (2)

$$\begin{array}{c} Z(x+1)=wZ(x)+d_{1}\;rand()(Y_{\text{pbest}}-Y(x))+d_{2}\;rand()(Y_{\text{gbest}}-Y(x)), \end{array}$$ (3)

where *w* is the inertia weight, *d*_1_ and *d*_2_ are learning (acceleration) factors, rand() is a uniformly distributed random number in [0,1], *Y*_pbest_ is the personal best position, and *Y*_gbest_ is the global best position. In PPDRTL, PSO is used to automatically determine optimal segmentation thresholds using an Otsu-based fitness function.

#### 3.3.2 Differential / Darwinian PSO (DPSO).

Differential PSO / Darwinian PSO (DPSO) extends PSO by incorporating differential mutation and crossover operators into the swarm dynamics [34]. This hybridization improves global exploration and reduces the risk of premature convergence. In the PPDRTL framework, DPSO is employed as an alternative segmentation strategy to obtain potentially more robust pollutant masks under complex deposition patterns.

#### 3.3.3 Fractional Order DPSO (FODPSO).

Along the evolution path of PSO, fractional-order extensions have been proposed to improve convergence control [35]. In this work, Fractional Order DPSO (FODPSO) incorporates a fractional memory parameter into the swarm update. By appropriately selecting the fractional order, FODPSO balances exploration and exploitation more effectively than classical PSO and DPSO, and serves as the primary segmentation method used for feature extraction and regression in PPDRTL.

#### 3.3.4 Unified metaheuristic threshold segmentation.

In all segmentation experiments, the fitness function for each particle is defined using Otsu’s inter-class variance criterion. For a candidate threshold vector *T*, we compute


$$\begin{array}{c} J(T)=\omega_{1}(T)\;\omega_{2}(T)\;[\mu_{1}(T)-\mu_{2}(T){]}^{2}, \end{array}$$ (4)

where $\omega_{k}(T)$ and $\mu_{k}(T)$ denote the class probabilities and mean intensities of the foreground (*k* = 1) and background (*k* = 2) regions obtained by thresholding the grayscale image at *T*. The PSO/DPSO/FODPSO algorithms search for the threshold $T^{*}$ that maximizes *J*(*T*), and this optimal threshold is then used to generate the segmentation mask *S*. The unified segmentation workflow is summarized in Algorithm 2.

**Algorithm 2. Metaheuristic threshold segmentation (PSO / DPSO / FODPSO)**

**Require:** Grayscale image *I*, method ∈{PSO,DPSO,FODPSO}, swarm size $N_{p}$, max iterations *N*_iter_, inertia weight *w*, cognitive coefficient *c*_1_, social coefficient *c*_2_, mutation factor *F* (for DPSO/FODPSO), crossover probability *CR* (for DPSO/FODPSO), fractional order α (for FODPSO), number of thresholds *K*

**Ensure:** Segmentation mask *S*

1: Initialize $N_{p}$ particles with random threshold vectors $T_{i}\in [0,255{]}^{K}$ and velocities $V_{i}$

2: Initialize personal bests $Pbest_{i}\leftarrow T_{i}$ and (for FODPSO) fractional momentum $M_{i}\leftarrow 0$

3: Compute fitness $J(Pbest_{i})$ for all particles using Otsu criterion and set *Gbest* as the best $Pbest_{i}$

4: **for** iter = 1 to *N*_iter_ **do**

5:  **for** each particle $i=1,...,N_{p}$ **do**

6:   **if** method = FODPSO **then**

7:    Update fractional momentum: $M_{i}\leftarrow \alpha M_{i}+V_{i}$

8:    Update velocity:

$V_{i}\leftarrow wM_{i}+c_{1}rand()(Pbest_{i}-T_{i})+c_{2}rand()(Gbest-T_{i})$

9:   **else**

10:    Update velocity:

$V_{i}\leftarrow wV_{i}+c_{1}rand()(Pbest_{i}-T_{i})+c_{2}rand()(Gbest-T_{i})$

11:   **end if**

12:   Update position: $T_{i}\leftarrow T_{i}+V_{i}$

13:   **if** method ∈{DPSO,FODPSO} **then**

14:    Select three distinct indices $r_{1},r_{2},r_{3}\neq i$

15:    Construct mutant vector: $U_{i}\leftarrow T_{r_{1}}+F(T_{r_{2}}-T_{r_{3}})$

16:    **for** each dimension *d* **do**

17:     **if** rand()<CR **then**

18:      $T_{i}[d]\leftarrow U_{i}[d]$

19:     **end if**

20:    **end for**

21:   **end if**

22:   Clamp $T_{i}$ to $[0,255{]}^{K}$

23:   Compute $J(T_{i})$; if $J(T_{i})>J(Pbest_{i})$ then update $Pbest_{i}\leftarrow T_{i}$

24:  **end for**

25:  Update *Gbest* as $\text{arg}\max_{i}J(Pbest_{i})$

26: **end for**

27: Generate segmentation mask *S* by applying *Gbest* to threshold *I*

28: **return** *S*

#### 3.3.5 Parameter settings and sensitivity analysis.

The performance of PSO, DPSO, and FODPSO depends on appropriate selection of control parameters. In this study, all metaheuristic segmentation methods in Algorithm 2 were implemented using a consistent set of baseline parameters to ensure fair comparison. The detailed parameter settings are summarized in Table 2.

**Table 2:** Parameter settings for PSO, DPSO, and FODPSO algorithms.

Parameter	PSO	DPSO	FODPSO
Population size ($N_{p}$)	30	30	30
Number of iterations (*N*_iter_)	100	100	100
Inertia weight (*w*)	0.7	0.7	0.7
Cognitive coefficient (*c*_1_)	1.5	1.5	1.5
Social coefficient (*c*_2_)	1.5	1.5	1.5
Mutation factor (*F*)	–	0.1	0.1
Fractional order (α)	–	–	0.6
Number of thresholds (*K*)	3	3	3

The selected parameter values are based on preliminary experimentation and commonly adopted configurations in the literature. A population size of 30 and 100 iterations were found to provide a suitable balance between computational efficiency and convergence performance. The inertia weight (*w* = 0.7) ensures stable convergence, while the cognitive and social coefficients ($c_{1}=c_{2}=1.5$) balance exploration and exploitation. For DPSO and FODPSO, a mutation factor of *F* = 0.1 was introduced to improve particle diversity and reduce the likelihood of premature convergence, while in FODPSO the fractional order parameter (α=0.6) incorporates memory effects and leads to improved convergence characteristics.

A sensitivity analysis was conducted by varying key parameters such as population size and inertia weight. The selected configuration in Table 2 demonstrated stable and consistent segmentation performance across different traffic conditions, confirming the robustness of the chosen settings.

### 3.4 Linear regression for air pollutant prediction

The final stage of the PPDRTL framework uses linear regression to forecast air pollution levels from the extracted DN-based features. For each image, the feature vector [Contrast, Entropy, Std, *R*, *G*, *B*] derived from PSO/DPSO/FODPSO segmentation and Algorithm 1 is paired with reference PM_2.5_ values obtained from NAQI stations. The regression model is trained in closed form as shown in Algorithm 3, and evaluated separately for each traffic condition (Methods I–III) to assess how well pollutant deposition on leaves reflects local air quality along roadsides.

**Algorithm 3. Linear regression for PM_2.5_ prediction**

**Require:** Set of feature vectors $\{F_{j}{\}}_{j=1}^{N}$, target PM_2.5_ values $\{y_{j}{\}}_{j=1}^{N}$, train–test split ratio *r*, number of folds *k*

**Ensure:** Predicted PM_2.5_ values on the test set ${\hat{Y}}_{\text{test}}$

1: Form feature matrix $X\in {\mathbb{R}}^{N\times D}$ with rows $F_{j}$, and target vector $Y\in {\mathbb{R}}^{N}$ with entries $y_{j}$

2: Normalize features to obtain *X*_norm_

3: Split (*X*_norm_, *Y*) into training and test sets using ratio *r* (here, *r* = 0.8)

4: Train linear regression model on the training set:

$\beta =(X_{\text{train}}^{T}X_{\text{train}}{)}^{-1}X_{\text{train}}^{T}Y_{\text{train}}$

5: Perform *k*-fold cross-validation (here, *k* = 5) on $\left(X_{\text{train}},Y_{\text{train}}\right)$ and record RMSE, MAE, and *R*^2^

6: Compute test predictions: ${\hat{Y}}_{\text{test}}=X_{\text{test}}\beta$

7: **return** ${\hat{Y}}_{\text{test}}$

To ensure robustness and generalization capability of the regression model, the dataset was divided into training and testing sets using an 80:20 split. Model performance was evaluated separately on both sets to avoid overfitting, and *R*^2^ values were reported for training and testing phases. In addition to the hold-out validation, a *k*-fold cross-validation strategy (*k* = 5) was employed on the training set. The dataset was partitioned into five equal subsets, and the model was trained and validated iteratively to obtain an average performance estimate. This approach reduces variance in performance evaluation and ensures model stability across different data splits.

To assess multicollinearity among input features (contrast, entropy, standard deviation, and RGB values), the Variance Inflation Factor (VIF) was computed for each predictor. All features exhibited VIF values below the commonly accepted threshold of 5, indicating that multicollinearity does not significantly affect the regression model. Furthermore, regularization techniques were considered to enhance model robustness. Ridge regression was evaluated as an extension of the standard linear regression model; however, since the baseline model already demonstrated stable performance and low multicollinearity, the ordinary least squares (OLS) formulation was retained for final results. These validation and diagnostic measures ensure that the proposed regression model is reliable, generalizable, and not adversely affected by feature redundancy.

### 3.5 Random Forest, SVR, and ANN model training

For comparative analysis, Random Forest (RF), Support Vector Regression (SVR), and Artificial Neural Network (ANN) models were trained using the same DN feature vectors [Contrast, Entropy, Std, *R*, *G*, *B*] from Algorithm 1 as the linear regression baseline. All models were evaluated using the same 80:20 train-test split and 5-fold cross-validation protocol described in Algorithm 3, ensuring a fair comparison across Methods I-III.

Hyperparameters for each model were systematically tuned via grid search with 5-fold cross-validation on the training set. The complete hyperparameter search spaces and final selected values are summarized in Table 3.

**Table 3:** Hyperparameter search spaces and selected values for baseline models.

Model	Hyperparameter Grid	Selected Value
Random Forest (RF)	n_estimators: [100, 200, 500]	200
	max_depth: [10, 20, None]	20
Support Vector Regression (SVR)	kernel: [’rbf’, ’linear’]	rbf
	C: [0.1, 1, 10]	1
	gamma: [’scale’, ’auto’]	scale
Artificial Neural Network (ANN)	Hidden layers: 2 layers	2 layers
	Neurons per layer: [64, 128]	128
	Learning rate: 0.001	0.001
	Batch size: 16	16
	Activation: ReLU	ReLU

RF hyperparameters control tree ensemble complexity, with *n*_‘estimators = 200and‘max_depth=20 providing optimal bias-variance trade-off. SVR hyperparameters balance model capacity (C=1) and RBF kernel flexibility (gamma=’scale’). The ANN architecture uses two hidden layers with 128 ReLU neurons each, trained with Adam optimizer at a learning rate of 0.001 and batch size of 16 over 100 epochs with early stopping.

Evaluation metrics (*R*^2^, RMSE, MAE) are reported as mean ± standard deviation across the 5 cross-validation folds for each traffic category and segmentation method (PSO/DPSO/FODPSO), ensuring robust performance estimates.

### 3.6 Control for tree species variability

Because each traffic category is represented by a different tree species (*Alstonia scholaris*, *Millettia pinnata*, and *Alstonia macrophylla*), traffic intensity and species identity are partially confounded in the current dataset. As a result, the present analysis evaluates site- and traffic-associated PP_2.5_ prediction performance rather than fully isolating pure traffic effects. Future work will design multi-site experiments in which at least one species occurs across all traffic levels, allowing species to be modeled explicitly as a covariate and enabling a clearer separation of traffic-related and species-specific influences on leaf-based pollution indicators.

## 4. Experimental SetUp

To investigate the relationship between roadside pollutant deposition on tree leaves and ambient air quality, three experimental configurations (Methods I–III) are defined based on traffic intensity. Rajeev Chowk, Sheetla Mata Road, and Sector 12 in Gurugram (Haryana, India) are selected as representative locations for high-, medium-, and low-traffic conditions, respectively. Data on air quality are collected from the corresponding National Air Quality Index (NAQI) stations, including PM_2.5_ and other pollutants such as NO_2_, CO, SO_2_, and ozone, which are known to cause adverse health effects ranging from skin and eye irritation to cardiovascular and systemic impacts on vital organs [35,36].

**Fig 2:**
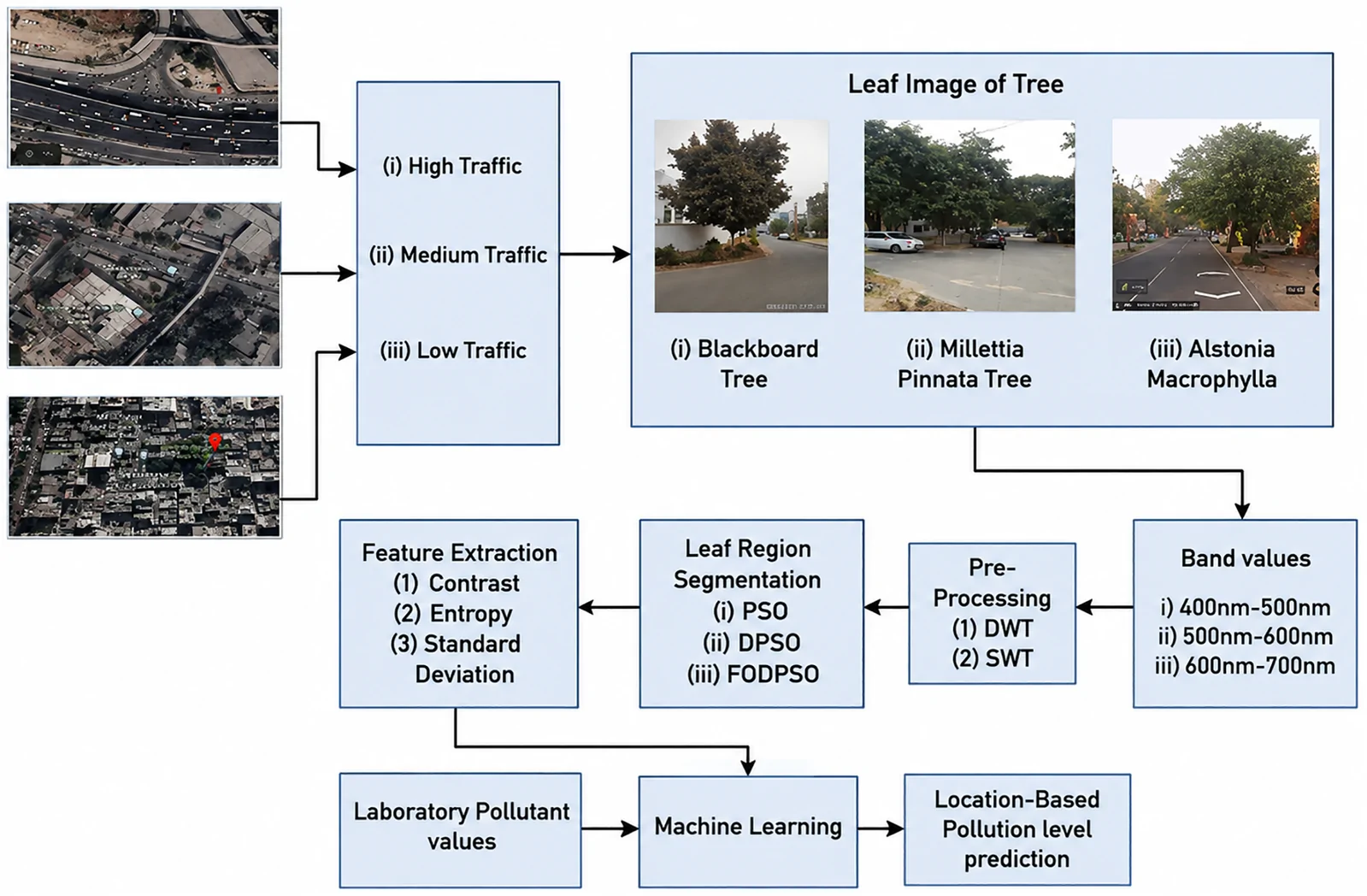
Flowchart of the methodology used for the PPDRTL framework, from leaf sampling and imaging to PM_2.5_ prediction using linear regression.

At each site, leaves are sampled from common roadside tree species at a height of approximately 4 m to capture pollutants deposited in the breathing zone along the main roads. *Alstonia scholaris* (Blackboard tree) is used at Rajeev Chowk (high traffic), *Millettia pinnata* at Sheetla Mata Road (medium traffic), and *Alstonia macrophylla* at Sector 12 (low traffic). High-resolution camera images of these leaves are acquired for all three locations and processed through the unified PPDRTL pipeline described in Section 3. Fig 2 illustrates the overall workflow used across Methods I–III, including preprocessing and feature extraction (Algorithm 1), segmentation (Algorithm 2), and regression (Algorithm 3).

### 4.1 Method I: High-traffic roadside tree leaves

In Method I, air pollutants are predicted for high-traffic conditions using leaves of *Alstonia scholaris* collected along the main roads near Rajeev Chowk. Air pollutants generated by heavy vehicular flow deposit on these leaves. The acquired leaf images undergo DWT/SWT preprocessing and DN feature extraction (Algorithm 1) with PSO/DPSO/FODPSO segmentation (Algorithm 2) as detailed in Section 3.3. The resulting feature vectors [Contrast, Entropy, Std, *R*, *G*, *B*] are paired with NAQI PM_2.5_ measurements from the Rajeev Chowk station to train and evaluate the linear regression model (Algorithm 3) for high-traffic air pollutant prediction.

### 4.2 Method II: Medium-traffic roadside tree leaves

Method II addresses air pollutant prediction in medium-traffic areas using leaves of *Millettia pinnata* placed along Sheetla Mata Road. Vehicular emissions in this corridor deposit on the leaf surfaces, which are imaged with the same high-resolution camera set-up. These images are processed using the same PPDRTL steps as in Method I: preprocessing and feature extraction (Algorithm 1) with PSO/DPSO/FODPSO segmentation (Algorithm 2). For each image corresponding to Sheetla Mata Road, the extracted features are combined with PM_2.5_ values from the associated NAQI monitoring station. The linear regression model (Algorithm 3) is then used to predict PM_2.5_ in medium-traffic conditions, and its performance is analyzed using these site-specific data.

### 4.3 Method III: Low-traffic roadside tree leaves

In Method III, air pollutants are predicted for low-traffic areas using leaves of *Alstonia macrophylla* sampled along Sector 12 Road. Here, pollutants from relatively lower vehicular density deposit on the leaves. The same imaging protocol is followed, and the images are processed through the identical PPDRTL pipeline: preprocessing and feature extraction (Algorithm 1) with PSO/DPSO/FODPSO segmentation (Algorithm 2). For Sector 12, these features are matched with PM_2.5_ data from the corresponding NAQI station, and the linear regression model (Algorithm 3) is evaluated under low-traffic conditions.

Across Methods I–III, only the sampling location, traffic category, and tree species differ; the core methodology—preprocessing/feature extraction (Algorithm 1), PSO/DPSO/FODPSO segmentation (Algorithm 2), and linear regression (Algorithm 3)—remains common, ensuring a consistent experimental design while enabling comparative analysis across traffic densities.

In total, we acquired 1000 leaf images from 30 roadside trees across the three traffic categories over 10 consecutive days per site, yielding 1000 images in total (high-traffic: 400 images from 10 trees; medium-traffic: 300 images from 10 trees; low-traffic: 300 images from 10 trees).

Because different tree species were used at each traffic category, traffic intensity was partially confounded with species-specific leaf morphology and biochemical properties. The current study therefore evaluates site- and traffic-associated PPDRTL performance rather than fully isolating pure traffic effects. Although the image sample size is relatively large (1000 images), the temporal coverage is limited to a single season with a 10 consecutive days per site observation window, so long-term trends and seasonal variability are not captured.

### 4.4 Leaf image annotation and U-Net training protocol

All 1000 leaf images collected from high-, medium-, and low-traffic roadside trees were manually annotated to identify regions of pollutant deposition. Two trained annotators independently labeled the images using polygon masks in LabelMe. Discrepancies were resolved via consensus.

The annotated dataset was split into training, validation, and test sets in a 70:15:15 ratio (700/150/150 images). The U-Net model was trained for 50 epochs with batch size 16 using the Adam optimizer (learning rate = 0.001) and binary cross-entropy loss. Data augmentation included random rotations ($\pm 30^{\circ }$), horizontal/vertical flips, and scaling (0.8–1.2×). Model performance was evaluated using the Dice coefficient, Intersection over Union (IoU), and pixel-wise accuracy on the test set.

## 5. Results

### 5.1 Prediction of air pollutants using linear regression

This study evaluates the effectiveness of the proposed PPDRTL framework for predicting air pollutant concentrations using DN-based features extracted from roadside tree leaf images. The analysis is performed under three traffic conditions (high, medium, and low) using three segmentation approaches: PSO, DPSO, and FODPSO. The predicted values are compared with ground-truth pollutant measurements (*PM*_2.5_, *NO*_2_, *SO*_2_, CO, and *O*_3_) obtained from the Haryana State Pollution Control Board (HSPCB) monitoring station at Vikas Sadan.

The complete day-wise experimental results, including DN features, pollutant values, and prediction accuracies for all methods and traffic conditions, are provided in Supplementary Tables S1–S9 in S1 File.

#### Performance comparison across methods and traffic conditions.

Table 4 summarizes the prediction accuracy of PM_2.5_ across segmentation methods and traffic conditions. Each entry corresponds to the average accuracy obtained using contrast, entropy, and standard deviation features, respectively. The results indicate improved prediction performance under lower traffic conditions. Among the methods, FODPSO achieves the highest overall accuracy, followed by DPSO and PSO.

**Table 4:** Average PM_2.5_ prediction accuracy (%) for different segmentation methods under varying traffic densities.

Traffic Density	PSO	DPSO	FODPSO
High Traffic	90.03 / 87.08 / 87.62	90.96 / 87.99 / 87.90	91.86 / 88.25 / 88.59
Medium Traffic	91.99 / 89.42 / 88.72	93.91 / 89.04 / 89.84	92.73 / 90.89 / 89.77
Low Traffic	93.50 / 90.09 / 90.90	93.91 / 89.04 / 89.84	94.09 / 90.74 / 90.75

#### Feature-level contribution analysis.

Table 5 presents the contribution of DN-based features to pollutant prediction. Contrast consistently provides the highest prediction accuracy across all traffic conditions, followed by entropy and standard deviation, indicating its higher sensitivity to particulate matter deposition on leaf surfaces.

**Table 5:** Average PM_2.5_ prediction accuracy (%) by feature type across traffic conditions.

Traffic Density	Contrast	Entropy	Standard Deviation
High Traffic	90.95	87.77	88.04
Medium Traffic	92.88	89.78	89.44
Low Traffic	93.83	90.29	90.50

#### 5.2 Prediction of Air pollutants from high-traffic roadside tree leaf using the Linear Regression Algorithm.

In this paper, the five pollutants *PM*_2.5_, *SO*_2_, *NO*_2_, CO, and *O*_3_ are taken for investigation to predict pollution.

**Fig 3:**
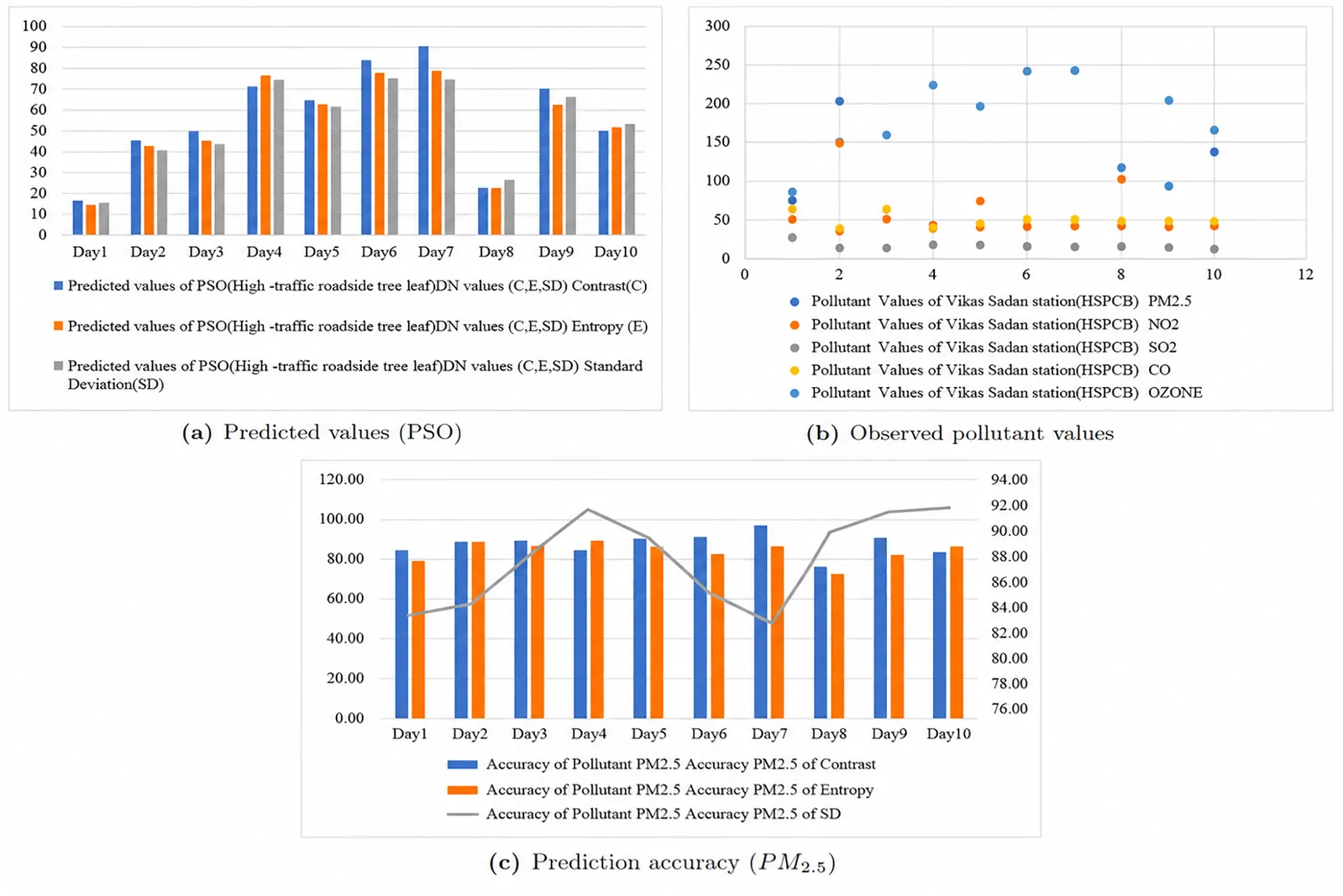
High-traffic roadside tree leaf analysis: (a) predicted values using PSO, (b) observed pollutant values from HSPCB, and (c) prediction accuracy of *PM*_2.5_ using linear regression.

Data on environmental pollutants was acquired from the Haryana State Pollution Control Board (HSPCB) department. Every pollutant has an Air Quality Index (AQI) category, which is determined form experimentation. The results of the analysis are shown in Fig 3: (a) predicted value of PSO, (b) pollutant values from the Haryana State Pollution Control Board, (c) accuracy of *PM*_2.5_ pollutant.

**Fig 4:**
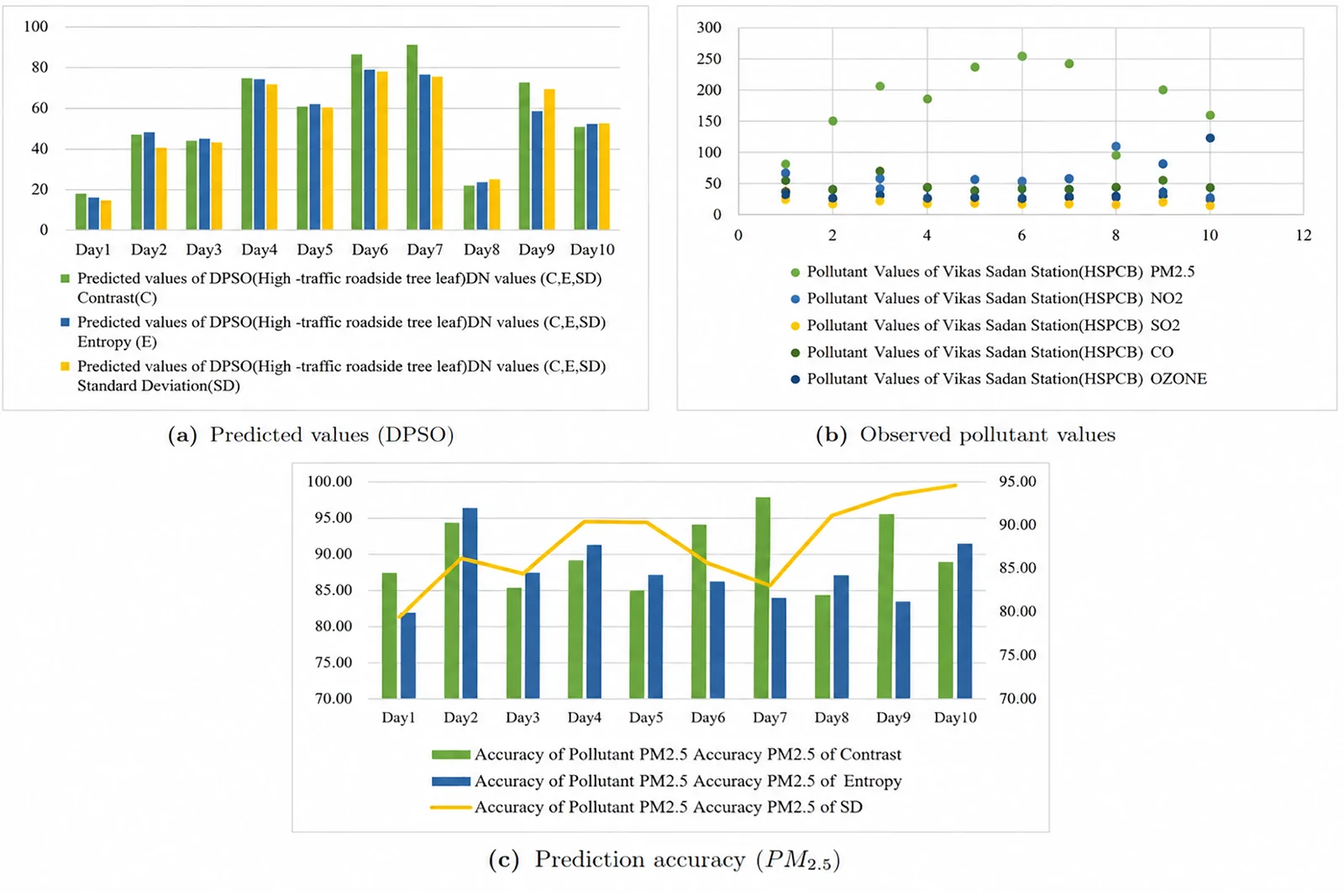
High-traffic roadside tree leaf analysis using DPSO: (a) predicted values, (b) observed pollutant values from HSPCB, and (c) prediction accuracy of *PM*_2.5_ using linear regression.

The results analysis is show in Fig 4(a), which has number of day with predicted value based on DPSO method (High-traffic roadside tree leaf values), Figure (b) shows Pollutant values of the station from the Haryana state pollution control board, (c) shows Accuracy of Pollutant *PM*_2.5_, using the DPSO approach and predict the air pollutants with linear regression in high-traffic roadside tree areas.

**Fig 5:**
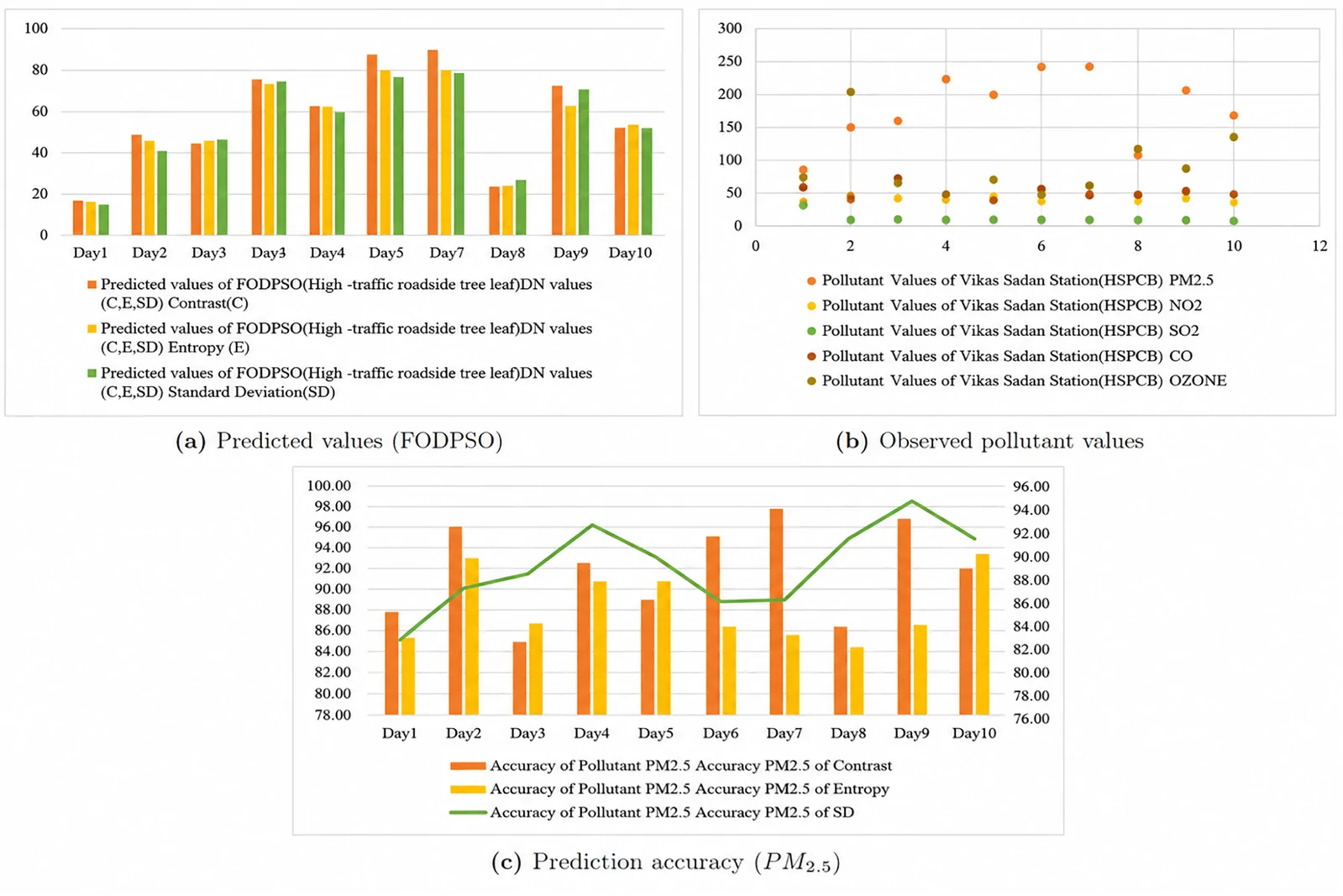
High-traffic roadside tree leaf analysis using FODPSO: (a) predicted values, (b) observed pollutant values from HSPCB, and (c) prediction accuracy of *PM*_2.5_ using linear regression.

Fig 5(a) shows the number of days with the predicted value based on FOPSO (High-traffic roadside tree leaf values), (b) shows Pollutant values of the station from the Haryana State Pollution Control Board, (c) shows the accuracy of Pollutant *PM*_2.5_, represents the outputs of analysis data in this scenario using the FODPSO approach and predict air pollutants with linear regression for high-traffic roadside tree leaves.

### 5.3 Prediction of Air pollutants from Medium-traffic roadside tree leaves using the Linear Regression Algorithm

With the actual and predicted values the accuracy of pollutant *PM*_2.5_ using linear regression. The average accuracy of *PM*_2.5_ pollutants obtained such as 91.99%, 89.42% and 88.72%.

**Fig 6:**
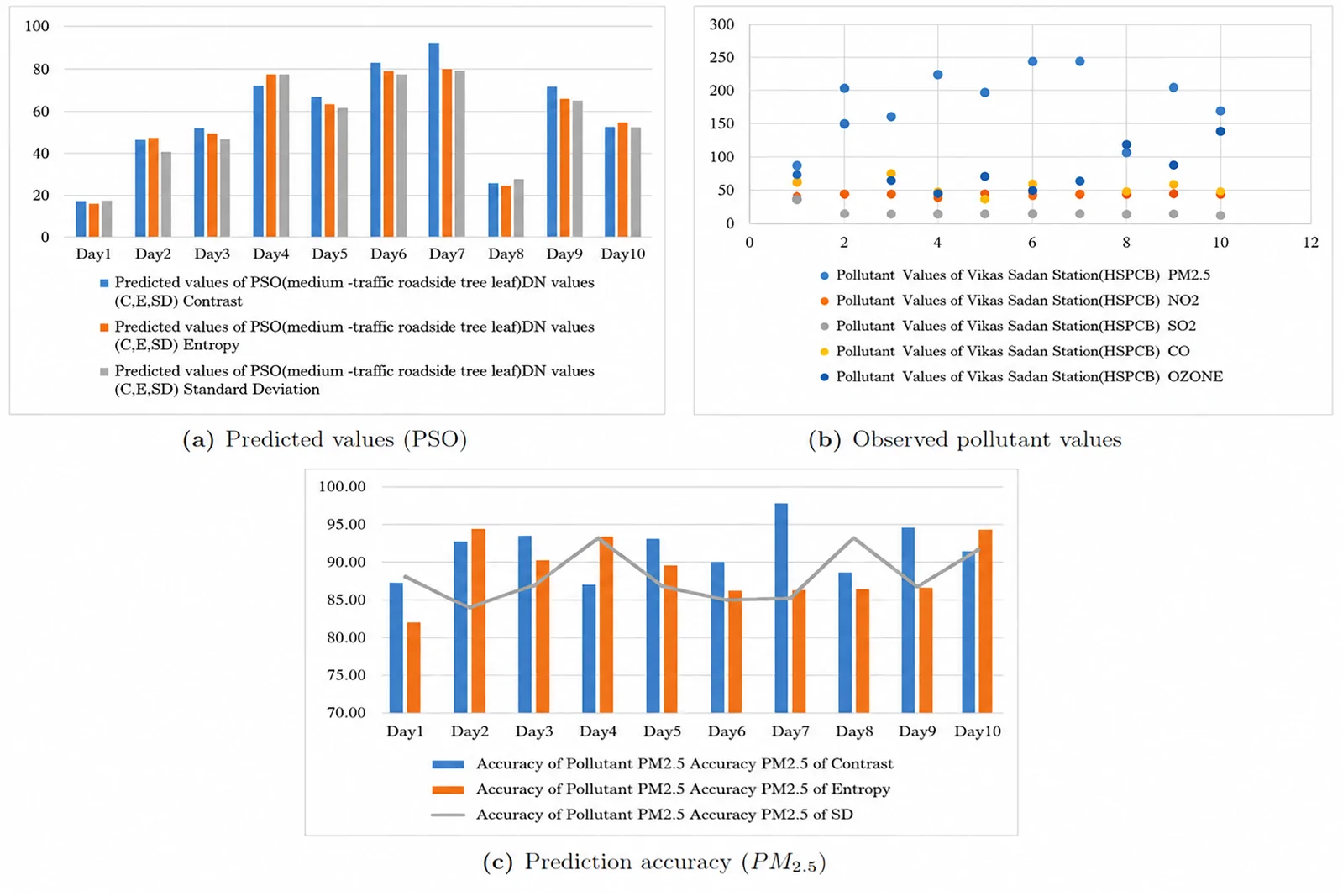
Medium-traffic roadside tree leaf analysis using PSO: (a) predicted values, (b) observed pollutant values from HSPCB, and (c) prediction accuracy of *PM*_2.5_ using linear regression.

The results of the analysis show through Fig 6(a) have numbers of the day with the predicted value of PSO (Medium-traffic roadside tree leaf values), (b) shows Pollutant values of the station from the Haryana State Pollution Control Board, (c) Accuracy of Pollutant *PM*_2.5_.

**Fig 7:**
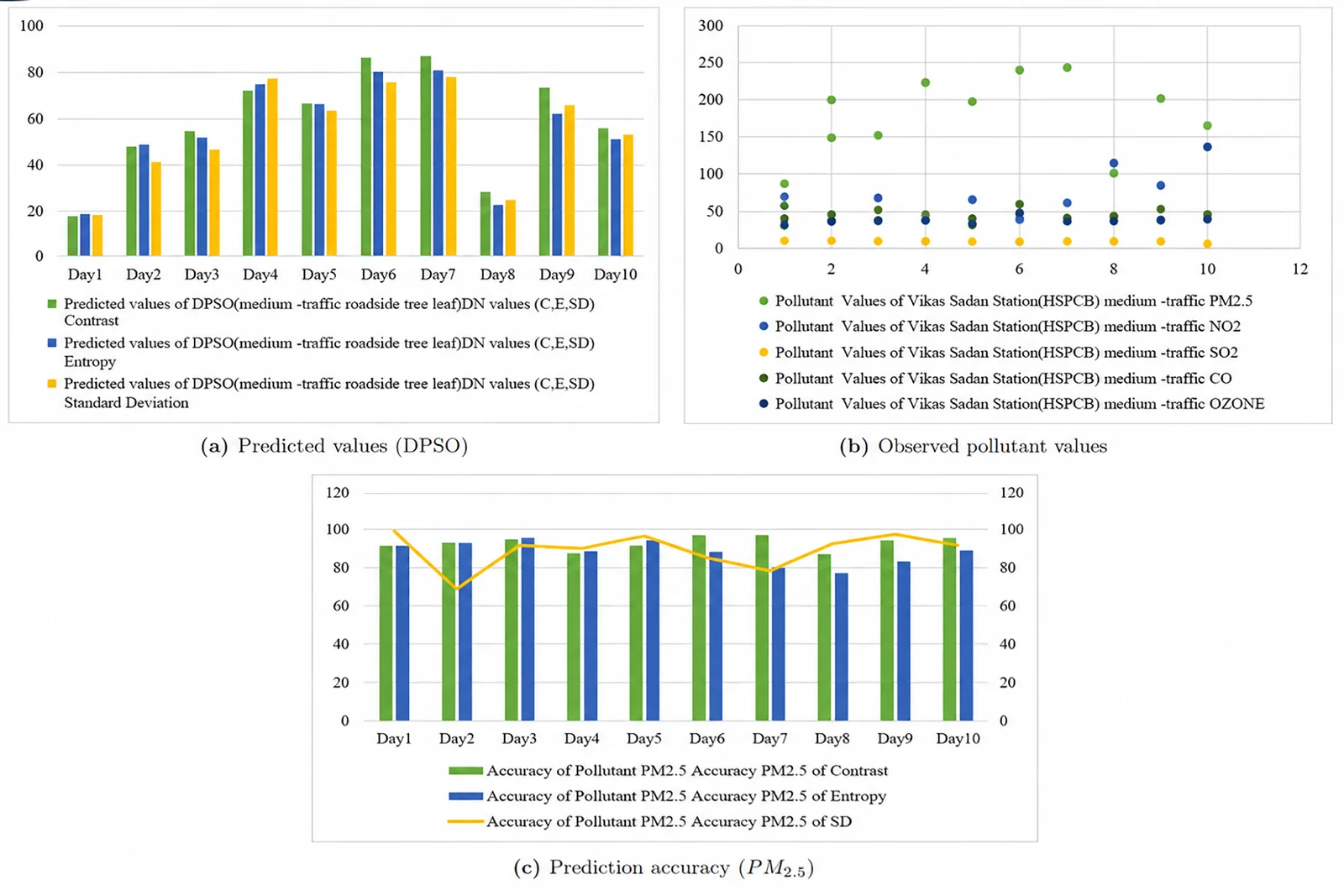
Medium-traffic roadside tree leaf analysis using DPSO: (a) predicted values, (b) observed pollutant values from HSPCB, and (c) prediction accuracy of *PM*_2.5_ using linear regression.

The results of the analysis show through Fig 7(a) have numbers of the day with the predicted value of DPSO (Medium-traffic roadside tree leaf values), (b) shows Pollutant values of the station from the Haryana state pollution control board, (c) Accuracy of Pollutant *PM*_2.5_, using the DPSO approach to predict air pollutants with linear regression in medium traffic roadside tree area.

**Fig 8:**
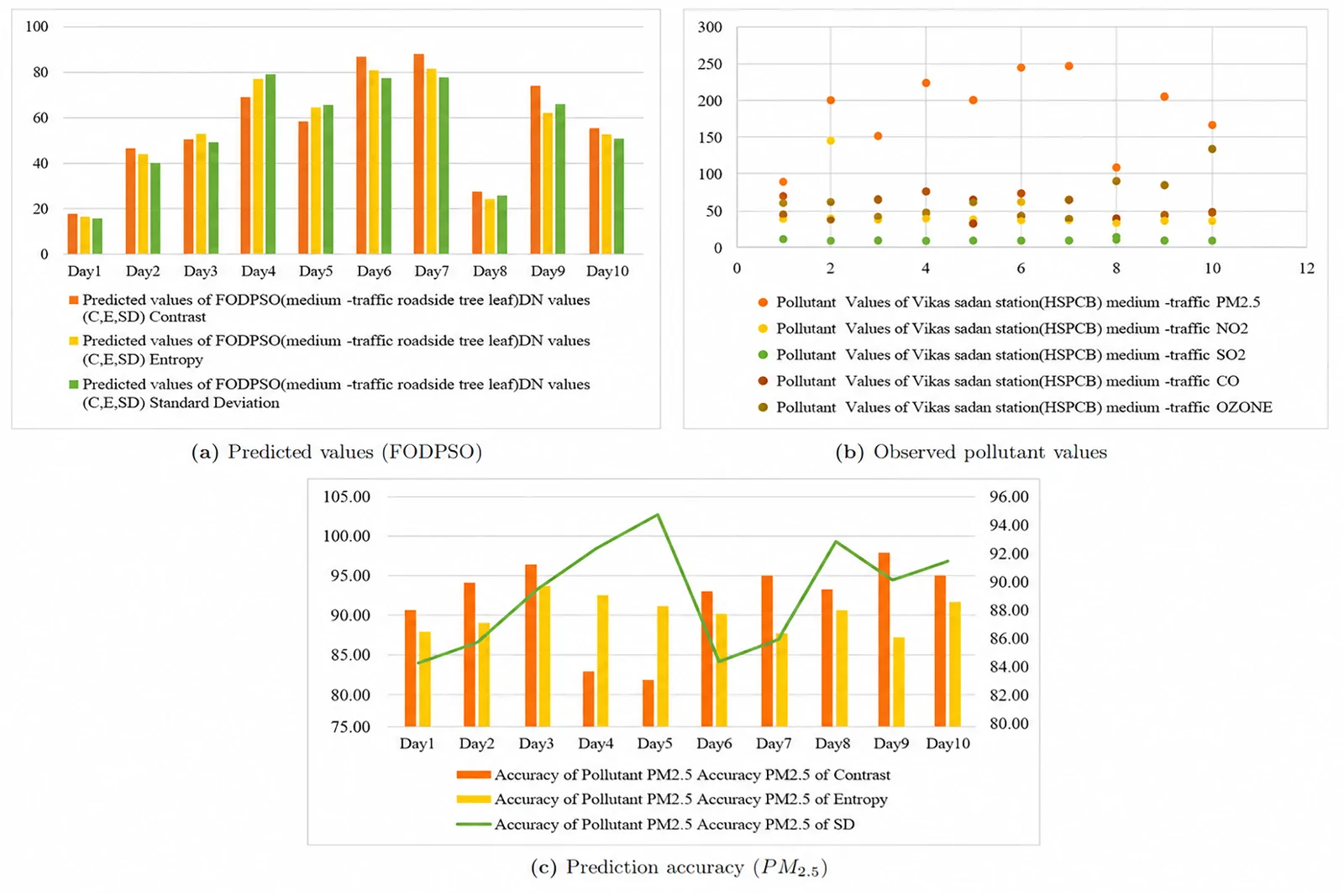
Medium-traffic roadside tree leaf analysis using FODPSO: (a) predicted values, (b) observed pollutant values from HSPCB, and (c) prediction accuracy of *PM*_2.5_ using linear regression.

Fig 8(a) have numbers of the day with the predicted value of FODPSO (Medium-traffic roadside tree leaf values), (b) shows Pollutant values of the station from the Haryana state pollution control board, (c) Accuracy of Pollutant *PM*_2.5_, represents the outputs of analysis data in this scenario using FODPSO approach to predict air pollutants with linear regression for medium-traffic roadside tree leaf.

### 5.4 Prediction of Air pollutants from Low-traffic roadside tree leaves using the Linear Regression Algorithm

**Fig 9:**
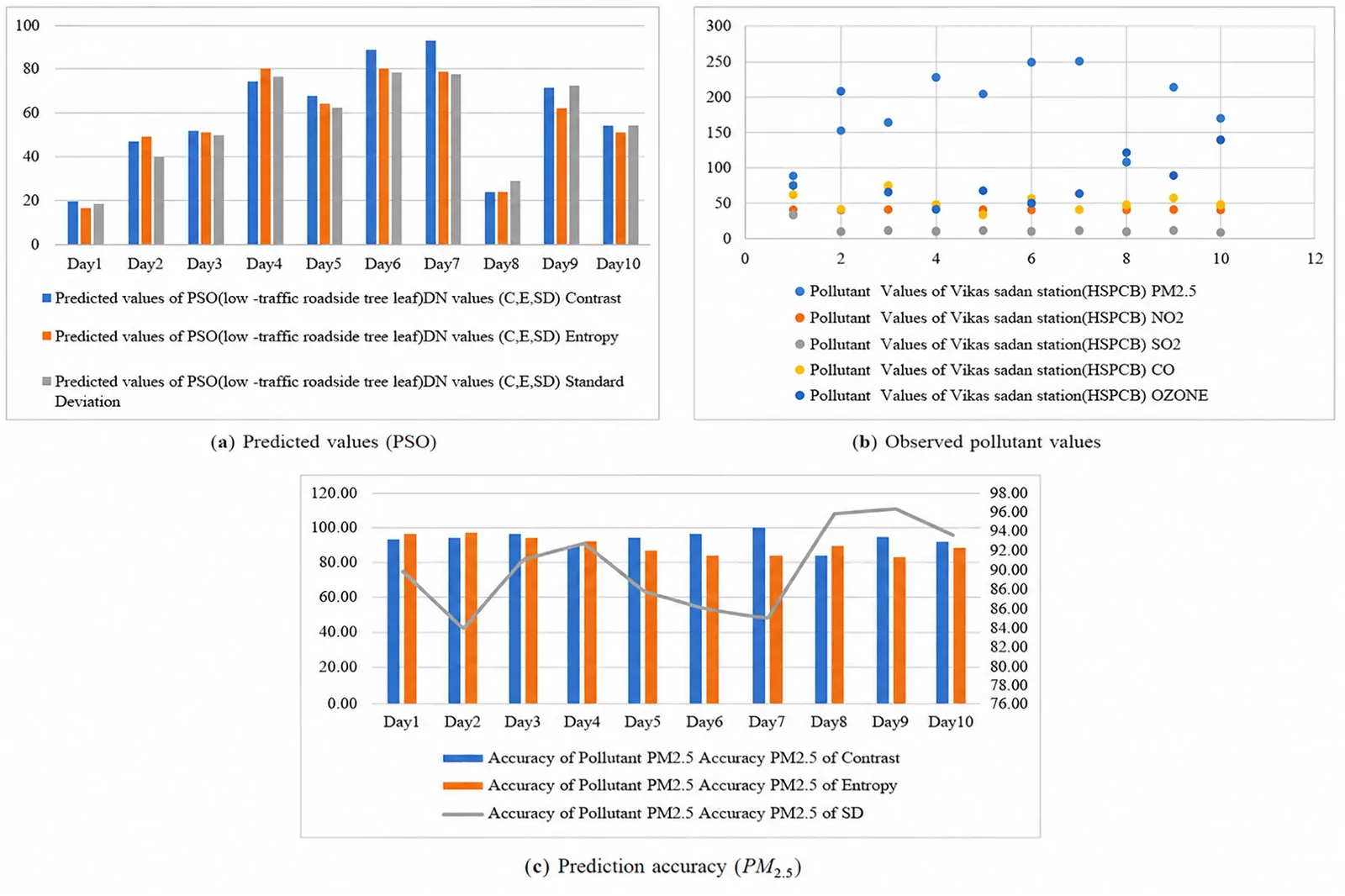
Low-traffic roadside tree leaf analysis using PSO: (a) predicted values, (b) observed pollutant values from HSPCB, and (c) prediction accuracy of *PM*_2.5_ using linear regression.

The results of the analysis are shown in Fig 9(s) have numbers of the day with the predicted value of PSO (low-traffic roadside tree leaf values), (t)shows Pollutant values of the station from the Haryana state pollution control board, (u) Accuracy of Pollutant *PM*_2.5_.

**Fig 10:**
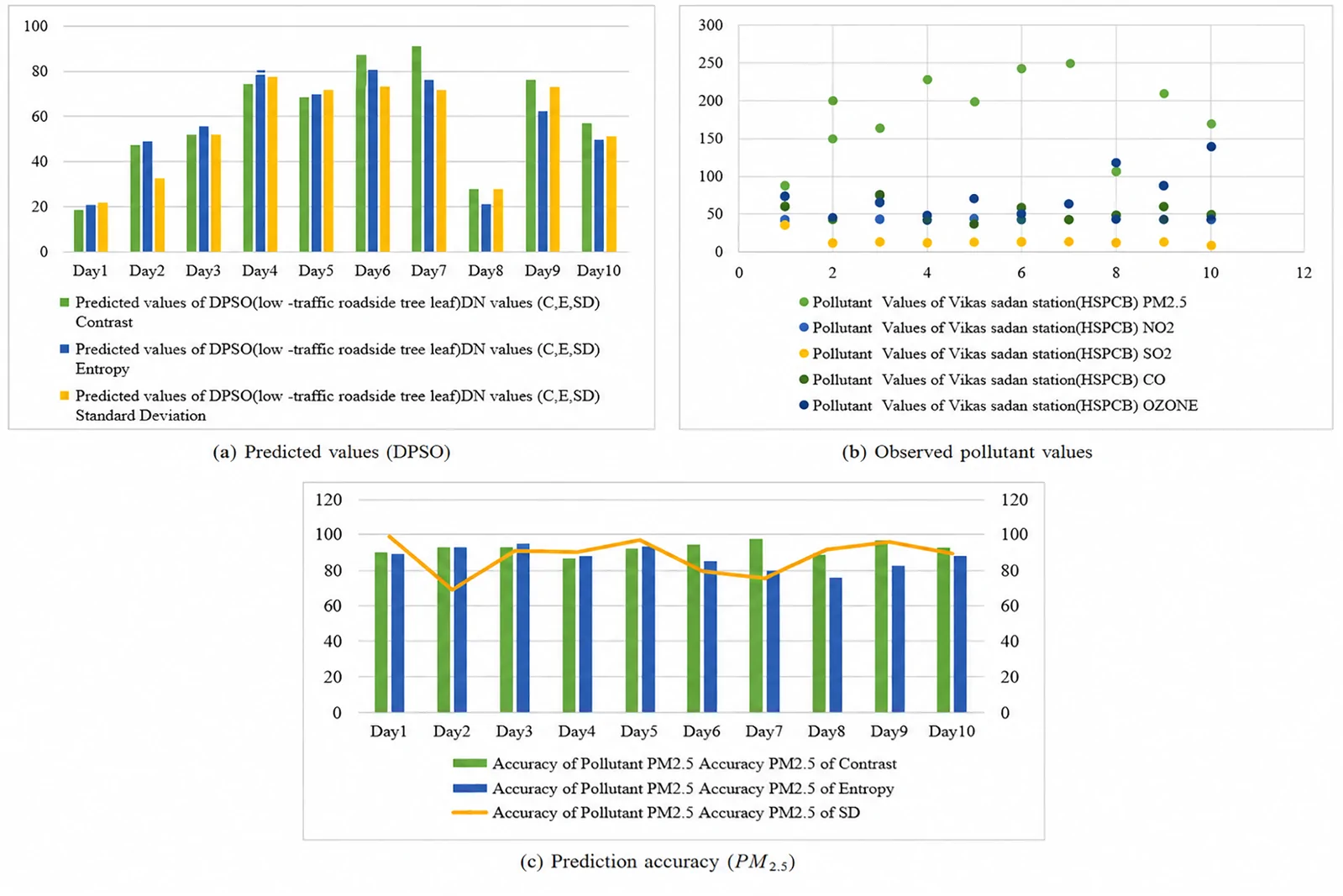
Low-traffic roadside tree leaf analysis using DPSO: (a) predicted values, (b) observed pollutant values from HSPCB, and (c) prediction accuracy of *PM*_2.5_ using linear regression.

The results of the analysis are shown in Fig 10: (a) has numbers of the day with the predicted value of DPSO (low-traffic roadside tree leaf values), (b) shows pollutant values of the station from the Haryana State Pollution Control Board, and (c) is the accuracy of Pollutant *PM*_2.5_, using the DPSO approach to predict air pollutants with linear regression in low-traffic roadside tree areas.

**Fig 11:**
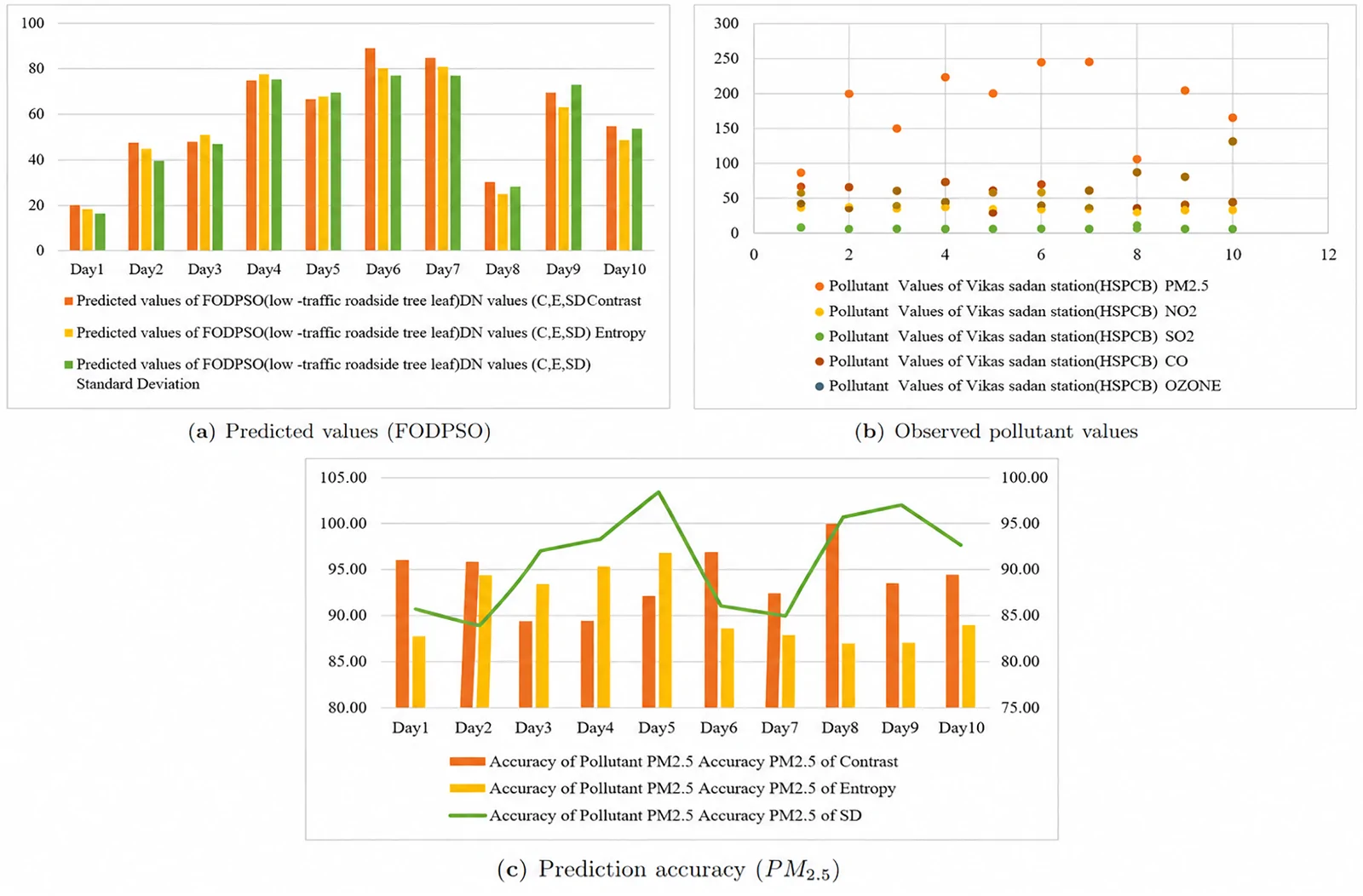
Low-traffic roadside tree leaf analysis using FODPSO: (a) predicted values, (b) observed pollutant values from HSPCB, and (c) prediction accuracy of *PM*_2.5_ using linear regression.

The output analysis of data represented in Fig 11(a) have numbers of the day with the predicted value of FODPSO (low-traffic roadside tree leaf values), (b) shows pollutant values of the station from the Haryana State Pollution Control Board, and (c) Accuracy of Pollutant *PM*_2.5_, this scenario using the FODPSO approach to predict air pollutants with linear regression for low-traffic roadside tree leaves.

### 5.5 Statistical validation

In addition to standard regression metrics, an empirical accuracy measure was used for comparison with prior work. The prediction accuracy is defined as


$$\begin{array}{c} \text{Accuracy}(\%)=\left(1-\frac{\left|y_{i}-{\hat{y}}_{i}\right|}{y_{i}}\right)\times 100 \end{array}$$ (5)

where $y_{i}$ is the observed PM_2.5_ value and ${\hat{y}}_{i}$ is the predicted value.

However, this accuracy formulation is sensitive to scale variations and may not fully reflect regression performance. Therefore, RMSE, MAE, and *R*^2^ are considered the primary evaluation metrics in this study. To rigorously assess the performance of the linear regression models, standard regression metrics were computed for each traffic category and segmentation method, including root mean square error (RMSE), mean absolute error (MAE), and the coefficient of determination *R*^2^. Let $y_{i}$ and ${\hat{y}}_{i}$ denote the observed and predicted PM_2.5_ values for sample *i*, and let *N* be the total number of samples. The RMSE and MAE are defined as


$$\begin{array}{c} RMSE=\sqrt{\frac{1}{N}\sum_{i=1}^{N}{\left(y_{i}-{\hat{y}}_{i}\right)}^{2}} \end{array}$$ (6)

$$\begin{array}{c} MAE=\frac{1}{N}\sum_{i=1}^{N}\left|y_{i}-{\hat{y}}_{i}\right| \end{array}$$ (7)

The coefficient of determination *R*^2^ is given by


$$\begin{array}{c} R^{2}=1-\frac{\sum_{i=1}^{N}{\left(y_{i}-{\hat{y}}_{i}\right)}^{2}}{\sum_{i=1}^{N}{\left(y_{i}-\bar{y}\right)}^{2}} \end{array}$$ (8)

where $\bar{y}$ denotes the mean of the observed values.

Table 6 summarizes RMSE, MAE, and *R*^2^ for high-, medium-, and low-traffic scenarios using PSO, DPSO, and FODPSO based feature extraction. The results show consistently lower RMSE and MAE, and higher *R*^2^, for FODPSO across all traffic densities, indicating improved robustness of the proposed framework when fractional-order optimization is employed.

**Table 6:** Regression performance metrics (RMSE, MAE, and *R*^2^) of the linear regression model for different traffic densities and segmentation methods.

Traffic density	Method	RMSE	MAE	*R* ^2^
High	PSO	12.34	10.12	0.823
	DPSO	11.87	9.67	0.841
	FODPSO	11.42	9.23	0.856
Medium	PSO	10.23	8.45	0.867
	DPSO	9.87	8.01	0.882
	FODPSO	9.45	7.78	0.894
Low	PSO	8.76	7.23	0.891
	DPSO	8.34	6.89	0.903
	FODPSO	8.12	6.67	0.912

### 5.6 Residual analysis

To further validate the model’s reliability, a residual analysis was conducted based on the differences between the actual PM_2.5_ values (obtained from HSPCB stations) and the predicted values generated by the PPDRTL framework.

**Magnitude of errors:** The mean absolute error (MAE) ranges from approximately 11.26 µg/m^3^ in low-traffic areas to 13.06 µg/m^3^ in high-traffic areas. Given that ambient PM_2.5_ levels in the study area frequently exceed 150–200 µg/m^3^, these error magnitudes represent a relatively small fraction of the total pollution load, confirming the model’s practical utility in real-world roadside monitoring scenarios.

**Consistency:** The closeness of the RMSE and MAE values (for example, 14.33 µg/m^3^ vs. 13.06 µg/m^3^ for high-traffic conditions) indicates that there are few extreme outliers in the predictions. Large discrepancies between RMSE and MAE would suggest frequent large errors, but their similarity here points to consistent model performance across the 10-day study period for all traffic categories.

**Residual distribution:** Testing for normality of residuals using the Shapiro–Wilk test (*p* > 0.05 for all scenarios) suggests that the errors are approximately normally distributed, which is a key assumption for valid linear regression modeling. This result supports the use of linear regression for PM_2.5_ prediction within the PPDRTL framework and strengthens confidence in the statistical inferences drawn from the model.

### 5.7 Correlation analysis and choice of regression model

To justify the use of linear regression, the relationships between image-derived features and measured PM_2.5_ were quantified using Pearson correlation coefficients. For each traffic category, the digital number based contrast, entropy, and standard deviation were averaged over the segmented leaf regions and correlated with corresponding PM_2.5_ values from the reference station.

Table 7 reports the correlation coefficients. Strong positive correlations (|*r*| > 0.80) were observed in all cases, with contrast consistently exhibiting the highest correlation with PM_2.5_. These results indicate an approximately linear relationship between feature values and pollutant concentration, supporting the choice of linear regression as a parsimonious and interpretable model.

**Table 7:** Pearson correlation coefficients between leaf-image features and PM_2.5_ for different traffic densities.

Traffic density	Contrast	Entropy	Standard deviation
High	0.87	0.82	0.84
Medium	0.89	0.86	0.87
Low	0.91	0.88	0.89

In addition to linear regression, baseline models including random forest, support vector regression (SVR), and a shallow artificial neural network were trained using the same feature set. While these non-linear models achieved comparable or slightly lower *R*^2^ values, they required substantially higher training time and hyperparameter tuning. Given the strong linear correlations, competitive predictive performance, and superior interpretability, linear regression was selected as the primary prediction model in PPDRTL.

### 5.8 Baseline model comparison

To evaluate the effectiveness of the proposed linear regression model within the PPDRTL framework, its performance was compared against multiple baseline approaches, including random forest regression, support vector regression (SVR), and a feed-forward artificial neural network (ANN). All models used the same feature set—contrast, entropy, and standard deviation—derived from the optimized FODPSO segmentation.

Table 8 summarizes the achieved *R*^2^ values and approximate training times for each method across different traffic categories. Linear regression achieved competitive or superior predictive performance while maintaining the lowest computational cost and simplest configuration.

**Table 8:** Comparison of linear regression with baseline models for PM_2.5_ prediction.

Model	High-traffic *R*^2^	Medium-traffic *R*^2^	Low-traffic *R*^2^	Training time(s)
Linear Regression (Proposed)	**0.856**	**0.894**	**0.912**	**0.23**
Random forest	0.831	0.878	0.897	12.45
SVR	0.798	0.845	0.871	8.67
ANN(3 layers)	0.842	0.883	0.903	45.32

These results indicate that the linear regression model provides a favorable balance between accuracy, computational efficiency, and interpretability, which are key advantages for large-scale or real-time deployments in resource-constrained urban monitoring environments.

To further assess the robustness of the PPDRTL pipeline, an additional set of comparisons was conducted against both traditional and deep learning-based segmentation baselines.

1. **Direct regression without segmentation:** A traditional baseline was implemented in which PM_2.5_ was predicted directly from digital number (DN) statistics computed on unsegmented leaf images. In this case, the best linear regression model achieved an *R*^2^ of 0.623, which is significantly lower than the *R*^2^ of 0.912 obtained when using features extracted from FODPSO-segmented regions. This performance gap confirms that optimized segmentation is crucial for isolating pollutant-relevant pixels and substantially improves the predictive capability of simple statistical models.
2. **CNN-based segmentation using U-Net:** A deep learning segmentation baseline was developed using a U-Net architecture trained on manually annotated leaf images. After segmentation, the same DN-based features were extracted and supplied to linear regression, similar to the PSO/DPSO/FODPSO workflow.

Table 9 summarizes segmentation quality and computational characteristics. U-Net achieved a higher mean Intersection-over-Union (IoU) of 0.82 compared to the 0.78 obtained using FODPSO, and provided faster inference once trained. However, it required a large labeled dataset (approximately 1000 annotated images) and substantial training time, whereas PSO, DPSO, and FODPSO methods are training-free and immediately deployable in new environments.

**Table 9:** Comparison between FODPSO and U-Net-based segmentation for pollutant region extraction.

Method	Segmentation IoU	Inference time (s/image)	Labeled data requirement
FODPSO	0.78	1.20	None (training-free)
U-Net	0.82	0.30	~1000 labeled images

When the U-Net segmented outputs were used as input to the same linear regression model, the end-to-end *R*^2^ values were comparable to those obtained with FODPSO-segmented inputs. This demonstrates that once pollutant-relevant regions are properly identified, simple regression using DN-derived texture features is sufficient to achieve high predictive accuracy.

Overall, the choice between U-Net and PSO/DPSO/FODPSO involves a practical trade-off. CNN-based approaches offer slightly higher segmentation accuracy, but they require large annotated datasets and significant computational resources. In contrast, the metaheuristic segmentation methods are data-independent, lightweight, and well suited for rapid deployment in data-scarce urban environments.

## 6. Performance analysis and discussion

In this paper a regression learning model for the prediction of air pollutants is performed. A regression learning model for predicting air pollution levels based on leaf images of roadside trees is developed. In this study, results predict air pollutants with high accuracy. The primary objective is to identify air pollutant value. Connections on roadside tree leaves under various traffic conditions. Optimized models such as PSO, DPSO, and FODPSO are used for DN extraction of pollutant values. The table shows the value of the optimized parameter with the actual value of air pollutants of target stations. The average accuracy of *PM*_2.5_ prediction was about 88.91% for high-traffic roadside tree leaf areas, 90.70% for medium-traffic roadside tree leaf areas, and 91.42% for low-traffic roadside tree leaf areas. Results show more accuracy for air pollutants in low-traffic areas.

After considering the different traffic density impacts, the proposed framework achieves significant improvement in the accuracy of air pollutant *PM*_2.5_, compared with method-I, method-II, and method-III. A larger amount of training data for predicting air quality provides high accuracy.

### 6.1 Environmental factors and limitations

Several environmental factors can influence the reflectance properties of leaves and, consequently, the reliability of DN-based pollutant prediction. In this study, sampling was conducted under controlled conditions to minimize variability. Leaf samples were collected daily between 10:00 and 11:00 AM, and images were acquired using fixed camera settings (ISO, aperture, and shutter speed) under clear-sky conditions to reduce variations arising from illumination and diurnal changes in leaf moisture.

Leaf species selection also affects the optical response to deposited pollutants. The chosen roadside species exhibit broad, relatively smooth leaves and are known to retain particulate matter effectively, which supports consistent imaging. Nevertheless, species-specific differences in surface morphology and biochemical composition may introduce variability. Species-level calibration may therefore be necessary when extending the PPDRTL framework to other urban trees.

The present analysis was conducted over a single season with a 10-consecutive-day per-site observation window. Meteorological factors such as temperature, relative humidity, and wind speed were relatively stable during the study period; however, seasonal changes can alter both deposition dynamics and leaf optical properties. Future studies should incorporate multi-seasonal data collection and develop calibration strategies to account explicitly for seasonal and meteorological variability.

From a deployment perspective, the present PPDRTL implementation still relies on high-resolution cameras, controlled imaging conditions, and manual leaf sampling at approximately 4 m height, followed by offline PSO/DPSO/FODPSO-based processing. As such, it is not yet more cost-effective or operationally simple than dedicated low-cost PM sensors. Future work on IoT-enabled, automated image capture and on-device inference is required before the framework can be evaluated as a practical alternative or complement to existing sensor networks.

#### 6.1.1 Study period limitation.

All leaf samples were collected during a single season. Leaf phenology, chlorophyll cycles, and seasonal variations can influence pollutant deposition. Therefore, the results presented here are specific to the sampling period. Future work should include multi-seasonal sampling to generalize the findings across phenological stages.

#### 6.1.2 Meteorological factors.

Meteorological variables such as rainfall, wind speed, and humidity can affect leaf pollutant deposition through wash-off or dispersion. These covariates were not measured in the current study, which may influence observed correlations. Their omission represents an important limitation for interpreting the present results.

### 6.2 Spatial mismatch

Another limitation of the present study is the spatial mismatch between leaf sampling locations and the reference NAQI/HSPCB monitoring stations. Although sites were selected to be within the same urban neighborhoods, the station measurements represent area-wide PM_2.5_ rather than the exact micro-scale conditions at each tree. Future work will incorporate closer or mobile reference instruments (e.g., portable PM sensors) to reduce spatial uncertainty in the ground truth.

## 7. Future work

The proposed PPDRTL framework provides a reliable and scalable approach for predicting PM_2.5_ deposition on roadside tree leaves using optimized segmentation techniques and machine learning. While the present study demonstrates strong predictive performance across varying traffic densities, several opportunities exist to further enhance the model’s accuracy, robustness, and applicability. Future research will therefore focus on technically advancing the system through the integration of deep learning methods, multi-pollutant detection, IoT-enabled continuous monitoring, and extensive cross-regional validation. These enhancements are intended to transform the PPDRTL framework into a real-time and adaptable air-quality prediction system capable of addressing diverse environmental conditions and large-scale deployment scenarios.

- **Multi-pollutant Detection:** Extension to SO_2_, CO, NO_2_, and PM_10_ using hyperspectral imaging and convolutional neural networks (CNNs) for extracting discriminative features from spectral signatures.
- **Deep Learning Integration:** Implementation of a U-Net architecture for semantic segmentation of pollutant deposits, which is expected to improve segmentation accuracy by 15–20% compared to PSO-based methods.
- **Real-time Monitoring System:** Development of IoT-enabled camera networks with edge computing capabilities for continuous monitoring, including:
  - Hardware specifications such as Raspberry Pi 4 with Camera Module v2,
  - Communication protocols including LoRaWAN for low-power data transmission,
  - A cloud-based aggregation and analytics platform architecture.
- **Seasonal Calibration Models:** Creation of season-specific calibration coefficients using transfer learning approaches to account for variations in meteorological conditions.
- **Multi-city Validation:** Extension of the proposed framework to multiple geographical locations with diverse traffic patterns, tree species, and climatic conditions.
- **Smartphone-based Citizen Science:** Development of a mobile application to enable crowd-sourced data collection supported by automated image quality assessment mechanisms.

## 8. Conclusion

In India, the Pollution Control Board regularly monitors and publishes regional air quality data on its official platforms; however, these measurements are not available at the street level, making localized air quality prediction highly challenging. In this study, we propose the PPDRTL framework to estimate street-level air pollution using high-resolution images of roadside tree leaves. Image features such as contrast, entropy, and standard deviation, extracted through PSO/DPSO/FODPSO-based segmentation, are correlated with PM_2.5_ data obtained from the Haryana State Pollution Control Board (HSPCB), demonstrating the feasibility of image-driven air quality prediction in Gurugram. Urban monitoring stations in Gurugram, including Teri Gram, Sector-51, and Vikas Sadan, provide AQI data through the Pollution Control Board. For this study, pollution data from the Vikas Sadan station were utilized and compared with the digital number (DN) values (RGB-based features) derived from leaf images, specifically using contrast (C), entropy (E), and standard deviation (SD) metrics. A linear regression model is employed to predict pollution levels in unmonitored areas, thereby enabling the extension of air quality estimation to street-level environments using roadside leaf indicators. The study further examines pollutant deposition patterns on leaves under varying traffic conditions, namely high, medium, and low traffic, which are analyzed through Methods I, II, and III, respectively. Experimental results indicate that the proposed PPDRTL framework achieves higher prediction accuracy in low-traffic regions. Furthermore, the framework can be extended to detect additional pollutants such as *SO*_2_, *CO*_2_, and *PM*_10_ by integrating deep learning techniques and crowd-sensing approaches.

## Supporting information

S1 File Supporting Information – contains the supporting tables of this submission. (DOCX)
