# Supplementary material for: PPDRTL: A novel framework for predicting pollutant deposition on roadside tree leaves using linear regression
Source: PLoS One. 2026 Sep 8;21(9):e0326588. doi: 10.1371/journal.pone.0326588 (PMC13552962; doi:10.1371/journal.pone.0326588)
Supplement: Supplementary file 1 [file pone.0326588.s001.docx]

9 **Supplementary Material**	1

Detailed day-wise results for PSO, DPSO, and FODPSO methods under high-,	2

medium-, and low-traffic conditions are presented in Tables S1-S9.	3

# 9.1 Prediction of Air Pollutants from High-Traffic Roadside 4

# Tree Leaves 5

Five pollutants (*PM*_2_*_._*_5_, *SO*_2_, *NO*_2_, CO, and *O*_3_) are considered for analysis, with	6

*PM*_2_*_._*_5_ as the primary focus.	7

Table S1 presents the comparison of predicted and observed pollutant levels for	8

high-traffic roadside tree leaves. Ground-truth pollutant data are obtained from the	9

pollution control board, while predicted values are derived from DN features (Contrast,	10

Entropy, and Standard Deviation) extracted from segmented leaf images using PSO,	11

DPSO, and FODPSO. Linear regression is applied to model the relationship between	12

leaf-based features and pollutant concentrations.	13

**Table S1.** Pollutant level and PSO value obtained from high-traffic roadside tree leaves

| **Day** | **Predicted values of**  **PSO(High -traffic roadside tree**  **leaf)DN values**  **(C,E,SD)** | | | **Pollutant Values of**  **Vikas Sadan Station (Haryana State Pollu-tion Control Board)** | | | | | **Accuracy of Pollu-**  **tant** *PM*_2_*_._*_5_ | | |
| --- | --- | --- | --- | --- | --- | --- | --- | --- | --- | --- | --- |
|  | **(C)** | **(E)** | **(SD)** | *PM*2*.*5 | *NO*_2_ | *SO*_2_ | *CO* | *O*_3_ | **Acc.**  *PM*2*.*5  **of Con-**  **trast** | **Acc.**  *PM*2*.*5  **of En-**  **tropy** | **Acc.**  *PM*2*.*5  **of SD** |
| **1** | 17 | 15 | 16 | 85 | 38 | 32 | 57 | 71 | 85.22 | 79.93 | 82.58 |
| **2** | 45 | 45 | 40 | 148 | 38 | 9 | 39 | 205 | 91.51 | 91.51 | 83.91 |
| **3** | 49 | 46 | 47 | 159 | 38 | 9 | 71 | 62 | 90.84 | 86.60 | 88.01 |
| **4** | 71 | 77 | 76 | 224 | 38 | 9 | 44 | 40 | 86.58 | 92.61 | 91.60 |
| **5** | 65 | 64 | 63 | 198 | 38 | 9 | 32 | 65 | 91.13 | 89.99 | 88.86 |
| **6** | 85 | 78 | 76 | 244 | 38 | 9 | 54 | 46 | 92.39 | 85.94 | 84.09 |
| **7** | 91 | 79 | 75 | 246 | 38 | 9 | 38 | 60 | 97.13 | 86.15 | 82.50 |
| **8** | 23 | 23 | 27 | 104 | 38 | 9 | 44 | 116 | 82.63 | 82.63 | 91.29 |
| **9** | 71 | 63 | 69 | 207 | 38 | 9 | 54 | 85 | 93.69 | 85.00 | 91.52 |
| **10** | 51 | 52 | 53 | 167 | 38 | 7 | 44 | 136 | 89.19 | 90.53 | 91.88 |
|  |  |  |  |  |  |  |  |  | 90.03 | 87.08 | 87.62 |

In the present analysis, the pollution levels obtained from the pollution board	14

department are used as the dependent variable (Y), and the tree leaf region based	15

pollution levels are the independent variable (X). Table S1 shows 10 days analysis of	16

predicted value from segmented leaves in high-traffic roadside tree digital number (DN)	17

of contrast(C), Entropy (E), and standard deviation(S). The lab value of air pollutants	18

from Vikas Sadan Station (HSPCB) is shown in Table S1. Lab and predicted values, the	19

accuracy of pollutant *PM*_2_*_._*_5_ using linear regression. The average accuracy of *PM*_2_*_._*_5_	20

pollutants obtained is about 90.03%, 87.08%and 87.62% respectively.	21

Table S2 represents the DPSO approach for the predicted data with the actual data	22

to calculate the accuracy of pollutants *PM*_2_*_._*_5_ using linear regression. The average	23

accuracy of *PM*_2_*_._*_5_ pollutants obtained was about 90.96%, 87.99% and 87.90%	24

respectively.	25

The average accuracy of *PM*_2_*_._*_5_ pollutant was about 91.86%, 88.25%, and 88.59%	26

respectively and shown in Table S3. Using FODPSO, predicted data of pollutant *PM*_2_*_._*_5_	27

| **Day** | **Predicted values**  **of DPSO(High**  **-traffic roadside tree leaf)DN val-**  **ues (C,E,SD)** | | | **Pollutant Values of**  **Vikas Sadan Sta-tion(HSPCB)** | | | | | **Accuracy of Pollutant**  *PM*2*.*5 | | |
| --- | --- | --- | --- | --- | --- | --- | --- | --- | --- | --- | --- |
|  | **(C)** | **(E)** | **(SD)** | *PM*2*.*5 | *NO*_2_ | *SO*_2_ | *CO* | *O*_3_ | **Acc.**  *PM*2*.*5  **of Con-**  **trast** | **Acc.**  *PM*2*.*5  **of En-**  **tropy** | **Acc.**  *PM*2*.*5  **of SD** |
| **1** | 18 | 16 | 15 | 85 | 38 | 32 | 57 | 71 | 87.87 | 82.58 | 79.93 |
| **2** | 47 | 48 | 42 | 148 | 38 | 9 | 39 | 205 | 94.55 | 96.07 | 86.95 |
| **3** | 46 | 47 | 45 | 159 | 38 | 9 | 71 | 62 | 86.60 | 88.01 | 85.18 |
| **4** | 75 | 76 | 74 | 224 | 38 | 9 | 44 | 40 | 90.60 | 91.60 | 89.59 |
| **5** | 61 | 62 | 64 | 198 | 38 | 9 | 32 | 65 | 86.59 | 87.72 | 89.99 |
| **6** | 87 | 79 | 78 | 244 | 38 | 9 | 54 | 46 | 94.24 | 86.86 | 85.94 |
| **7** | 92 | 77 | 77 | 246 | 38 | 9 | 38 | 60 | 98.04 | 84.33 | 84.33 |
| **8** | 24 | 25 | 27 | 104 | 38 | 9 | 44 | 116 | 84.80 | 86.96 | 91.29 |
| **9** | 73 | 62 | 70 | 207 | 38 | 9 | 54 | 85 | 95.86 | 83.91 | 92.60 |
| **10** | 52 | 53 | 54 | 167 | 38 | 7 | 44 | 136 | 90.53 | 91.88 | 93.23 |
|  |  |  |  |  |  |  |  |  | 90.96 | 87.99 | 87.90 |

using linear regression is shown in Table S3.	28

**Table S3.** Pollutant level and FODPSO value obtained from high-traffic roadside tree leaves

| **Day** | **Predicted values**  **of FODPSO(High**  **-traffic roadside tree leaf)DN val-**  **ues (C,E,SD)** | | | **Pollutant Values of**  **Vikas Sadan Sta-tion(HSPCB)** | | | | | **Accuracy of Pollu-**  **tant** *PM*_2_*_._*_5_ | | |
| --- | --- | --- | --- | --- | --- | --- | --- | --- | --- | --- | --- |
|  | **(C)** | **(E)** | **(SD)** | *PM*2*.*5 | *NO*_2_ | *SO*_2_ | *CO* | *O*_3_ | **Acc.**  *PM*2*.*5  **of Con-**  **trast** | **Acc.**  *PM*2*.*5  **of En-**  **tropy** | **Acc.**  *PM*2*.*5  **of SD** |
| **1** | 18 | 17 | 16 | 85 | 38 | 32 | 57 | 71 | 87.87 | 85.22 | 82.58 |
| **2** | 48 | 46 | 42 | 148 | 38 | 9 | 39 | 205 | 96.07 | 93.03 | 86.95 |
| **3** | 45 | 46 | 47 | 159 | 38 | 9 | 71 | 62 | 85.18 | 86.60 | 88.01 |
| **4** | 77 | 75 | 76 | 224 | 38 | 9 | 44 | 40 | 92.61 | 90.60 | 91.60 |
| **5** | 63 | 64 | 62 | 198 | 38 | 9 | 32 | 65 | 88.86 | 89.99 | 87.72 |
| **6** | 88 | 80 | 77 | 244 | 38 | 9 | 54 | 46 | 95.16 | 87.78 | 85.02 |
| **7** | 91 | 78 | 79 | 246 | 38 | 9 | 38 | 60 | 97.13 | 85.24 | 86.15 |
| **8** | 25 | 24 | 27 | 104 | 38 | 9 | 44 | 116 | 86.96 | 84.80 | 91.29 |
| **9** | 74 | 64 | 72 | 207 | 38 | 9 | 54 | 85 | 96.95 | 86.08 | 94.78 |
| **10** | 53 | 54 | 53 | 167 | 38 | 7 | 44 | 136 | 91.88 | 93.23 | 91.88 |
|  |  |  |  |  |  |  |  |  | 91.86 | 88.25 | 88.59 |

# 9.2 Prediction of Air pollutants from Medium-traffic roadside 29

# tree leaves using the Linear Regression Algorithm 30

Measured the air pollutant through leaf segmentation PSO in medium-traffic roadside	31

tree leaf image with the digital number (DN) of contrast(C), Entropy (E), and standard	32

deviation(S). The actual value of air pollutants of Vikas Sadan Station (HSPCB) is	33

shown in Table S4.	34

35

With the actual and predicted values the accuracy of pollutant *PM*_2_*_._*_5_ using linear	36

regression. The average accuracy of *PM*_2_*_._*_5_ pollutants obtained such as 91.99%,	37

89.42%and 88.72%.	38

| **Day** | **Predicted values**  **of PSO(medium**  **-traffic roadside tree leaf)DN val-ues (C,E,SD)** | | | **Pollutant Values of**  **Vikas Sadan Sta-tion(HSPCB)** | | | | | **Accuracy of**  **Pollutant** *PM*_2_*_._*_5_ | | |
| --- | --- | --- | --- | --- | --- | --- | --- | --- | --- | --- | --- |
|  | **(C)** | **(E)** | **(SD)** | *PM*2*.*5 | *NO*_2_ | *SO*_2_ | *CO* | *O*_3_ | **Acc.**  *PM*2*.*5  **of Con-**  **trast** | **Acc.**  *PM*2*.*5  **of En-**  **tropy** | **Acc.**  *PM*2*.*5  **of SD** |
| **1** | 18 | 16 | 18 | 85 | 38 | 32 | 57 | 71 | 87.87 | 82.58 | 87.87 |
| **2** | 46 | 47 | 41 | 148 | 38 | 9 | 39 | 205 | 93.03 | 94.55 | 85.43 |
| **3** | 51 | 49 | 47 | 159 | 38 | 9 | 71 | 62 | 93.67 | 90.84 | 88.01 |
| **4** | 72 | 78 | 78 | 224 | 38 | 9 | 44 | 40 | 87.58 | 93.61 | 93.61 |
| **5** | 67 | 64 | 62 | 198 | 38 | 9 | 32 | 65 | 93.40 | 89.99 | 87.72 |
| **6** | 83 | 79 | 78 | 244 | 38 | 9 | 54 | 46 | 90.55 | 86.86 | 85.94 |
| **7** | 92 | 80 | 79 | 246 | 38 | 9 | 38 | 60 | 98.04 | 87.07 | 86.15 |
| **8** | 26 | 25 | 28 | 104 | 38 | 9 | 44 | 116 | 89.13 | 86.96 | 93.45 |
| **9** | 72 | 65 | 65 | 207 | 38 | 9 | 54 | 85 | 94.78 | 87.17 | 87.17 |
| **10** | 53 | 55 | 53 | 167 | 38 | 7 | 44 | 136 | 91.88 | 94.57 | 91.88 |
|  |  |  |  |  |  |  |  |  | 91.99 | 89.42 | 88.72 |

Table S5 represents the approach DPSO for the predicted data with the actual data 39 to calculate the accuracy of pollutant *PM*_2_*_._*_5_ using linear regression for medium traffic 40 roadside tree leaves. The average accuracy of *PM*_2_*_._*_5_ pollutant obtained such as 93.91%, 41 89.04%and 89.84%.

**Table S5.** Pollutant level and DPSO value obtained from medium-traffic roadside tree leaves

| **Day** | **Predicted values**  **of DPSO(medium**  **-traffic roadside tree leaf)DN val-ues (C,E,SD)** | | | **Pollutant Values of**  **Vikas Sadan Sta-tion(HSPCB)** | | | | | **Accuracy of Pollu-**  **tant** *PM*_2_*_._*_5_ | | |
| --- | --- | --- | --- | --- | --- | --- | --- | --- | --- | --- | --- |
|  | **(C)** | **(E)** | **(SD)** | *PM*2*.*5 | *NO*_2_ | *SO*_2_ | *CO* | *O*_3_ | **Acc.**  *PM*2*.*5  **of Con-**  **trast** | **Acc.**  *PM*2*.*5  **of En-**  **tropy** | **Acc.**  *PM*2*.*5  **of SD** |
| **1** | 17 | 18 | 17 | 85 | 38 | 32 | 57 | 71 | 90.52 | 90.51 | 98.45 |
| **2** | 48 | 49 | 42 | 148 | 38 | 9 | 39 | 205 | 94.51 | 94.55 | 71.75 |
| **3** | 55 | 51 | 47 | 159 | 38 | 9 | 71 | 62 | 92.25 | 96.5 | 93.67 |
| **4** | 68 | 77 | 79 | 224 | 38 | 9 | 44 | 40 | 87.58 | 90.59 | 92.6 |
| **5** | 63 | 65 | 64 | 198 | 38 | 9 | 32 | 65 | 94.54 | 95.67 | 97.94 |
| **6** | 85 | 81 | 78 | 244 | 38 | 9 | 54 | 46 | 95.16 | 88.7 | 83.06 |
| **7** | 89 | 82 | 80 | 246 | 38 | 9 | 38 | 60 | 97.13 | 82.49 | 79.75 |
| **8** | 28 | 23 | 25 | 104 | 38 | 9 | 44 | 116 | 93.45 | 78.3 | 93.45 |
| **9** | 75 | 63 | 67 | 207 | 38 | 9 | 54 | 85 | 98.03 | 83.9 | 95.86 |
| **10** | 51 | 53 | 55 | 167 | 38 | 7 | 44 | 136 | 95.92 | 89.18 | 91.88 |
|  |  |  |  |  |  |  |  |  | 93.91 | 89.04 | 89.84 |

42

The average accuracy of *PM*_2_*_._*_5_ pollutant obtained such as 92.73%, 90.89%and	43

89.77% in Table S6. Using FODPSO for the predicted data with the actual data to	44

calculate the accuracy of pollutant *PM*_2_*_._*_5_ through linear regression technique for	45

medium traffic roadside tree leaf.	46

| **Day** | **Predicted values of**  **FODPSO(medium**  **-traffic roadside tree leaf)DN val-ues (C,E,SD)** | | | **Pollutant Values**  **of Vikas sadan sta-tion(HSPCB) medium**  **-traffic** | | | | | **Accuracy of Pollu-**  **tant** *PM*_2_*_._*_5_ | | |
| --- | --- | --- | --- | --- | --- | --- | --- | --- | --- | --- | --- |
|  | **(C)** | **(E)** | **(SD)** | *PM*2*.*5 | *NO*_2_ | *SO*_2_ | *CO* | *O*_3_ | **Acc.**  *PM*2*.*5  **of Con-**  **trast** | **Acc.**  *PM*2*.*5  **of En-**  **tropy** | **Acc.**  *PM*2*.*5  **of SD** |
| **1** | 19 | 18 | 17 | 85 | 38 | 32 | 57 | 71 | 90.52 | 87.87 | 85.22 |
| **2** | 47 | 45 | 42 | 148 | 38 | 9 | 39 | 205 | 94.55 | 91.51 | 86.95 |
| **3** | 53 | 51 | 49 | 159 | 38 | 9 | 71 | 62 | 96.50 | 93.67 | 90.84 |
| **4** | 69 | 77 | 79 | 224 | 38 | 9 | 44 | 40 | 84.57 | 92.61 | 94.62 |
| **5** | 58 | 68 | 66 | 198 | 38 | 9 | 32 | 65 | 83.18 | 94.54 | 92.27 |
| **6** | 86 | 84 | 77 | 244 | 38 | 9 | 54 | 46 | 93.32 | 91.47 | 85.02 |
| **7** | 90 | 82 | 80 | 246 | 38 | 9 | 38 | 60 | 96.22 | 88.90 | 87.07 |
| **8** | 28 | 27 | 28 | 104 | 38 | 9 | 44 | 116 | 93.45 | 91.29 | 93.45 |
| **9** | 76 | 62 | 68 | 207 | 38 | 9 | 54 | 85 | 99.13 | 83.91 | 90.43 |
| **10** | 56 | 54 | 53 | 167 | 38 | 7 | 44 | 136 | 95.92 | 93.23 | 91.88 |
|  |  |  |  |  |  |  |  |  | 92.73 | 90.89 | 89.77 |

# 9.3 Prediction of Air pollutants from Low-traffic roadside tree 47

# leaves using the Linear Regression Algorithm 48

Table S7 shows data analysis in which predicted value is measured through leaf	49

segmentation PSO in low-traffic roadside tree leaf images with the digital number (DN)	50

of contrast(C), Entropy (E), and standard deviation(S). And the actual value of air	51

pollutants of Vikas Sadan Station (HSPCB). With the actual and predicted values, the	52

accuracy of pollutant *PM*_2_*_._*_5_ using linear regression. The average accuracy of *PM*_2_*_._*_5_	53

pollutant obtained such as 93.50%, 90.09%and 90.90%.

**Table S7.** Pollutant level and PSO value obtained from low-traffic roadside tree leaves

| **Day** | **Predicted values of**  **PSO(low -traffic roadside tree**  **leaf)DN values**  **(C,E,SD)** | | | **Pollutant Values of**  **Vikas Sadan Station (HSPCB)** | | | | | **Accuracy of Pollu-**  **tant** *PM*_2_*_._*_5_ | | |
| --- | --- | --- | --- | --- | --- | --- | --- | --- | --- | --- | --- |
|  | **(C)** | **(E)** | **(SD)** | *PM*2*.*5 | *NO*_2_ | *SO*_2_ | *CO* | *O*_3_ | **Acc.**  *PM*2*.*5  **of Con-**  **trast** | **Acc.**  *PM*2*.*5  **of En-**  **tropy** | **Acc.**  *PM*2*.*5  **of SD** |
| **1** | 20 | 17 | 19 | 85 | 38 | 32 | 57 | 71 | 93.09 | 96.22 | 89.93 |
| **2** | 47 | 49 | 40 | 148 | 38 | 9 | 39 | 205 | 94.45 | 97.78 | 83.91 |
| **3** | 52 | 51 | 49 | 159 | 38 | 9 | 71 | 62 | 94.98 | 93.57 | 90.84 |
| **4** | 74 | 80 | 77 | 224 | 38 | 9 | 44 | 40 | 89.59 | 92.80 | 92.61 |
| **5** | 68 | 64 | 62 | 198 | 38 | 9 | 32 | 65 | 94.54 | 88.56 | 87.72 |
| **6** | 89 | 80 | 78 | 244 | 38 | 9 | 54 | 46 | 95.96 | 85.19 | 85.94 |
| **7** | 94 | 80 | 78 | 246 | 38 | 9 | 38 | 60 | 99.74 | 84.50 | 85.24 |
| **8** | 24 | 24 | 29 | 104 | 38 | 9 | 44 | 116 | 84.79 | 90.35 | 95.62 |
| **9** | 72 | 62 | 73 | 207 | 38 | 9 | 54 | 85 | 94.77 | 82.74 | 95.86 |
| **10** | 54 | 51 | 54 | 167 | 38 | 7 | 44 | 136 | 93.12 | 89.17 | 93.23 |
|  |  |  |  |  |  |  |  |  | 93.50 | 90.09 | 90.90 |

54

Table S8 represents the approach DPSO for the predicted data with the actual data	55

to calculate the accuracy of pollutant *PM*_2_*_._*_5_ using linear regression for low-traffic	56

roadside tree leaf. The average accuracy of *PM*_2_*_._*_5_ pollutant obtained such as 93.91%,	57

89.04%and 89.84%.	58

59

| **Day** | **Predicted values of**  **DPSO(low -traffic roadside tree**  **leaf)DN values**  **(C,E,SD)** | | | **Pollutant Values of**  **Vikas Sadan Station (HSPCB)** | | | | | **Accuracy of Pollu-**  **tant** *PM*_2_*_._*_5_ | | |
| --- | --- | --- | --- | --- | --- | --- | --- | --- | --- | --- | --- |
|  | **(C)** | **(E)** | **(SD)** | *PM*2*.*5 | *NO*_2_ | *SO*_2_ | *CO* | *O*_3_ | **Acc.**  *PM*2*.*5  **of Con-**  **trast** | **Acc.**  *PM*2*.*5  **of En-**  **tropy** | **Acc.**  *PM*2*.*5  **of SD** |
| **1** | 19 | 21 | 22 | 85 | 38 | 32 | 57 | 71 | 90.52 | 90.51 | 98.45 |
| **2** | 46 | 47 | 32 | 148 | 38 | 9 | 39 | 205 | 94.51 | 94.55 | 71.75 |
| **3** | 50 | 53 | 51 | 159 | 38 | 9 | 71 | 62 | 92.25 | 96.5 | 93.67 |
| **4** | 72 | 75 | 77 | 224 | 38 | 9 | 44 | 40 | 87.58 | 90.59 | 92.6 |
| **5** | 68 | 69 | 71 | 198 | 38 | 9 | 32 | 65 | 94.54 | 95.67 | 97.94 |
| **6** | 88 | 81 | 75 | 244 | 38 | 9 | 54 | 46 | 95.16 | 88.7 | 83.06 |
| **7** | 91 | 75 | 72 | 246 | 38 | 9 | 38 | 60 | 97.13 | 82.49 | 79.75 |
| **8** | 28 | 21 | 28 | 104 | 38 | 9 | 44 | 116 | 93.45 | 78.3 | 93.45 |
| **9** | 75 | 62 | 73 | 207 | 38 | 9 | 54 | 85 | 98.03 | 83.9 | 95.86 |
| **10** | 56 | 51 | 53 | 167 | 38 | 7 | 44 | 136 | 95.92 | 89.18 | 91.88 |
|  |  |  |  |  |  |  |  |  | 93.91 | 89.04 | 89.84 |

The average accuracy of PM_2_*_._*_5_ pollutants obtained is 94.09%, 90.74%and 90.75% in 60 Table S9. Using FODPSO for the predicted data with the actual data to calculate the 61 accuracy of pollutants *PM*_2_*_._*_5_ through the linear regression technique for low-traffic	62

roadside tree leaves.

**Table S9.** Pollutant level and FODPSO value obtained from low-traffic roadside tree leaves

| **Day** | **Predicted values**  **of FODPSO(low**  **-traffic roadside tree leaf)DN val-**  **ues (C,E,SD)** | | | **Pollutant Values of**  **Vikas Sadan Sta-tion(HSPCB)** | | | | | **Accuracy of Pollu-**  **tant** *PM*_2_*_._*_5_ | | |
| --- | --- | --- | --- | --- | --- | --- | --- | --- | --- | --- | --- |
|  | **(C)** | **(E)** | **(SD)** | *PM*2*.*5 | *NO*_2_ | *SO*_2_ | *CO* | *O*_3_ | **Acc.**  *PM*2*.*5  **of Con-**  **trast** | **Acc.**  *PM*2*.*5  **of En-**  **tropy** | **Acc.**  *PM*2*.*5  **of SD** |
| **1** | 21 | 18 | 17 | 85 | 38 | 32 | 57 | 71 | 95.81 | 87.87 | 85.22 |
| **2** | 48 | 47 | 40 | 148 | 38 | 9 | 39 | 205 | 96.07 | 94.55 | 83.91 |
| **3** | 48 | 51 | 49 | 159 | 38 | 9 | 71 | 62 | 89.43 | 93.67 | 90.84 |
| **4** | 74 | 80 | 77 | 224 | 38 | 9 | 44 | 40 | 89.59 | 95.62 | 92.61 |
| **5** | 68 | 69 | 71 | 198 | 38 | 9 | 32 | 65 | 92.27 | 95.68 | 97.95 |
| **6** | 90 | 81 | 78 | 244 | 38 | 9 | 54 | 46 | 97.00 | 88.70 | 85.94 |
| **7** | 86 | 81 | 78 | 246 | 38 | 9 | 38 | 60 | 92.56 | 87.98 | 85.24 |
| **8** | 31 | 25 | 29 | 104 | 38 | 9 | 44 | 116 | 99.94 | 86.96 | 95.62 |
| **9** | 71 | 65 | 73 | 207 | 38 | 9 | 54 | 85 | 93.69 | 87.17 | 96.95 |
| **10** | 55 | 51 | 54 | 167 | 38 | 7 | 44 | 136 | 94.57 | 89.19 | 93.23 |
|  |  |  |  |  |  |  |  |  | 94.09 | 90.74 | 90.75 |

63
